# Choosing Bad versus Worse: Predictions of Two-Photon-Absorption Strengths Based on Popular Density Functional Approximations

**DOI:** 10.1021/acs.jctc.1c01056

**Published:** 2022-01-26

**Authors:** Marta Chołuj, Md. Mehboob Alam, Maarten T. P. Beerepoot, Sebastian P. Sitkiewicz, Eduard Matito, Kenneth Ruud, Robert Zaleśny

**Affiliations:** † Faculty of Chemistry, Wrocław University of Science and Technology, Wyb. Wyspiańskiego 27, PL−50370 Wrocław, Poland; ‡ Department of Chemistry, Indian Institute of Technology Bhilai, Sejbahar, Raipur, Chhattisgarh 492015, India; § Hylleraas Centre for Quantum Molecular Sciences, Department of Chemistry, UiT The Arctic University of Norway, N-9037 Tromsø, Norway; ∥ Donostia International Physics Center (DIPC), Manuel Lardizabal Ibilbidea 4, 20018 Donostia, Euskadi, Spain; ⊥ Kimika Fakultatea, Euskal Herriko Unibertsitatea (UPV/EHU), 20080 Donostia, Euskadi, Spain; # Ikerbasque Foundation for Science, Plaza Euskadi 5, 48009 Bilbao, Euskadi, Spain

## Abstract

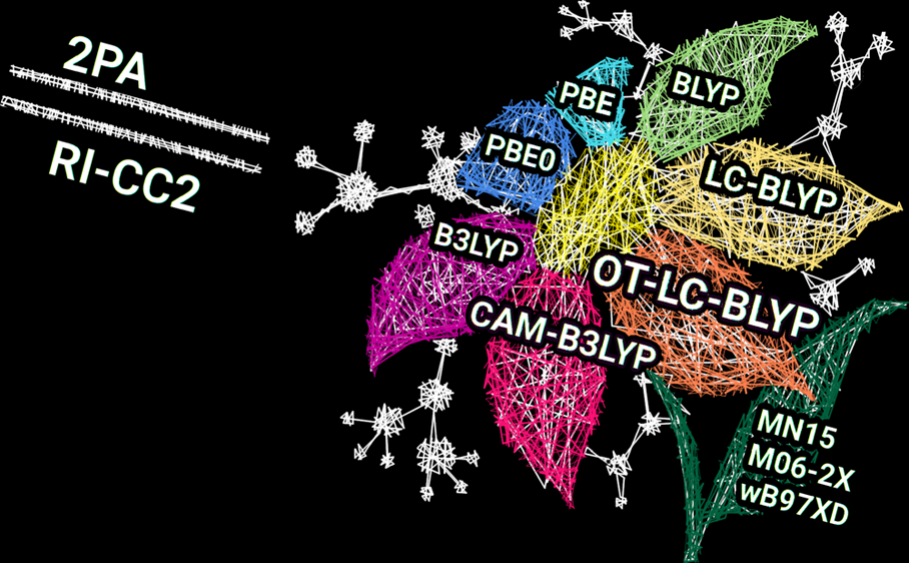

We present a benchmark study of density functional approximation (DFA) performances in predicting the two-photon-absorption strengths in π-conjugated molecules containing electron-donating/-accepting moieties. A set of 48 organic molecules is chosen for this purpose, for which the two-photon-absorption (2PA) parameters are evaluated using different DFAs, including BLYP, PBE, B3LYP, PBE0, CAM-B3LYP, LC-BLYP, and optimally tuned LC-BLYP. Minnesota functionals and ωB97X-D are also used, applying the two-state approximation, for a subset of molecules. The efficient resolution-of-identity implementation of the coupled-cluster CC2 model (RI-CC2) is used as a reference for the assessment of the DFAs. Two-state models within the framework of both DFAs and RI-CC2 are used to gain a deeper insight into the performance of different DFAs. Our results give a clear picture of the performance of the density functionals in describing the two-photon activity in dipolar π-conjugated systems. The results show that global hybrids are best suited to reproduce the absolute values of 2PA strengths of donor–acceptor molecules. The range-separated functionals CAM-B3LYP and optimally tuned LC-BLYP, however, show the highest linear correlations with the reference RI-CC2 results. Hence, we recommend the latter DFAs for structure–property studies across large series of dipolar compounds.

## 1 Introduction

Light–matter interactions have been the subject of intensive research both experimentally and theoretically for decades. The electric polarization of a molecular system exposed to a relatively weak electric field scales linearly with its amplitude and can be well described at the molecular level by the electric polarizability. However, in the presence of a strong electric field, generated, e.g., by a coherent intense light beam, nonlinear optical effects can be manifested. Depending on the specific nonlinear process, these can be described by first-, second-, or higher-order electric hyperpolarizabilities. One of the most widely studied nonlinear processes is the two-photon-absorption (2PA) phenomenon, in which two photons are simultaneously absorbed, leading to an excitation to a different state (rotational, vibrational, electronic, etc.). This phenomenon was theoretically predicted in 1931 by Maria Göppert-Mayer^1^ and subsequently experimentally verified in 1961 by Keiser and Garrett^2^ thanks to the invention of lasers.^3^ Nowadays, 2PA is an important and powerful spectroscopic tool, commonly used in photodynamic therapy,^4−6^ bioimaging,^7−11^ three-dimensional optical data storage,^12,13^ microfabrication,^14^ and two-photon lasing,^15^ to name a few areas of application. Technological advances trigger the quest for new materials with large 2PA cross sections. Studies in this field focus mainly on dipolar, quadrupolar, and octupolar organic dyes; dendrimers; nanoparticles; and metal–organic frameworks.^16−19^ Among the factors that significantly affect the 2PA response of molecular systems, one can highlight, *inter alia*, bond length alternation,^20,21^ solvent polarity,^22,23^ spatial confinement,^24−26^ and long-range charge-transfer processes.^27,28^ Gaining a thorough understanding of the relationship between molecular structure and optical response is of pivotal importance for designing new materials with desirable properties and exploring novel applications. Theoretical chemistry plays an important role in this area of research.^21,22,29−39^ Advanced electronic-structure calculations allow not only the optical properties of molecules and materials to be predicted accurately,^40^ but also allow the elucidation of results of experimental measurements. This holds in particular for the analysis of spectroscopic signatures in nonlinear absorption spectra. However, simulations of 2PA spectra can be challenging for computational chemistry, especially when relatively large chemical systems are considered. The methods used for simulations should not only predict excitation energies and 2PA cross sections with high accuracy but also account for many different factors that are important for bringing the results of the simulations closer to real experimental conditions, such as vibronic contributions and solvent effects.^7,22,33,35,41−49^

Over the past few decades, the potential of various computational approaches to predict 2PA spectra has been assessed.^50^ Response theory combined with coupled-cluster wave functions allows accurate 2PA spectra for a wide palette of chemically diverse compounds to be obtained.^51−53^ Unfortunately, this approach can quickly become computationally very expensive when applied to larger molecules (i.e., composed of dozens of atoms) and combined with the necessary split-valence triple- or quadruple-ζ basis sets. In such cases, density functional theory (DFT) constitutes an attractive alternative. However, selecting the density functional approximation (DFA) that ensures reliable results is by no means a trivial task. Therefore, taking into account the multitude of available DFAs, evaluating their performance in calculations of 2PA spectra against correlated wave function based methods is mandatory in order to employ DFT with confidence. Range-separated functionals (RSFs), with CAM-B3LYP at the forefront, are nowadays very often used to model 2PA spectra. These functionals include pure DFT exchange for short-range electron–electron interactions and the exact Hartree–Fock exchange for long-range interactions.^54,55^ Compared to many other DFAs, RSFs improve the accuracy of excitation energies to Rydberg and charge-transfer states.^56−61^ However, also RSFs can yield substantial errors for 2PA cross sections as has been demonstrated in several papers.^46,62−66^ Nevertheless, even though RSFs underestimate 2PA cross sections, the errors appear to be more systematic than those for semilocal and hybrid functionals. It should be noted that only a moderate number of organic molecules have been studied thus far with the use of different DFAs.

Improvements in the performance of RSFs in simulations can be achieved by a system-specific tuning of the range-separation parameter ω. Several techniques have been developed so far, adopting different constraints and targeting different molecular properties. Most commonly, the range-separation parameter is adjusted to impose Janak’s theorem,^67^ leading to optimally tuned RSFs (OT-RSFs).^68−70^ Numerous studies have reported that OT-RSFs perform very well for donor–acceptor systems and, compared to the standard versions of RSFs, provide an improved description of the energetics involving frontier orbitals, the position and intensity of one-photon-absorption bands in UV–vis spectra, and charge-transfer effects.^71−73^

Lin and Van Voorhis proposed triplet-tuned RSFs (TT-RSFs), in which ω is chosen to minimize the difference of the singlet–triplet excitation energy obtained with the ΔSCF and TD-DFT approaches.^74^ It was shown that TT-RSFs yield slightly superior singlet and triplet excitation energies compared with the ones obtained with OT-RSFs. For nonlinear optical phenomena, several studies have tested the applicability of the OT-based scheme. Most of those were limited to the computation of the static nonlinear optical properties, such as the static polarizability and first hyperpolarizability. The performance of OT-RSFs for computing those molecular properties is mixed and highly unsystematic, depending strongly on the size and chemical nature of the systems.^75−80^

Recently, Besalú-Sala, Luis, and co-workers have proposed a different type of tuning tailored for the computation of the static second hyperpolarizability, namely, the Tα-RSF scheme.^79^ In contrast to the OT- and TT-based approaches, it relies on the empirical correlation between the static polarizability (obtained with the default parametrization of the RSF) and the values of ω needed to reproduce CCSD(T) results for a collection of 60 molecular systems with a wide variety of first hyperpolarizabilities. While Tα-RSF reproduces the reference results with a remarkable precision for the broad variety of chemical systems studied, Tα-LC-BLYP has so far only been used to compute a single component of the second hyperpolarizability tensor, and its performance for rotationally averaged properties is therefore largely unknown.

**Scheme 1:**
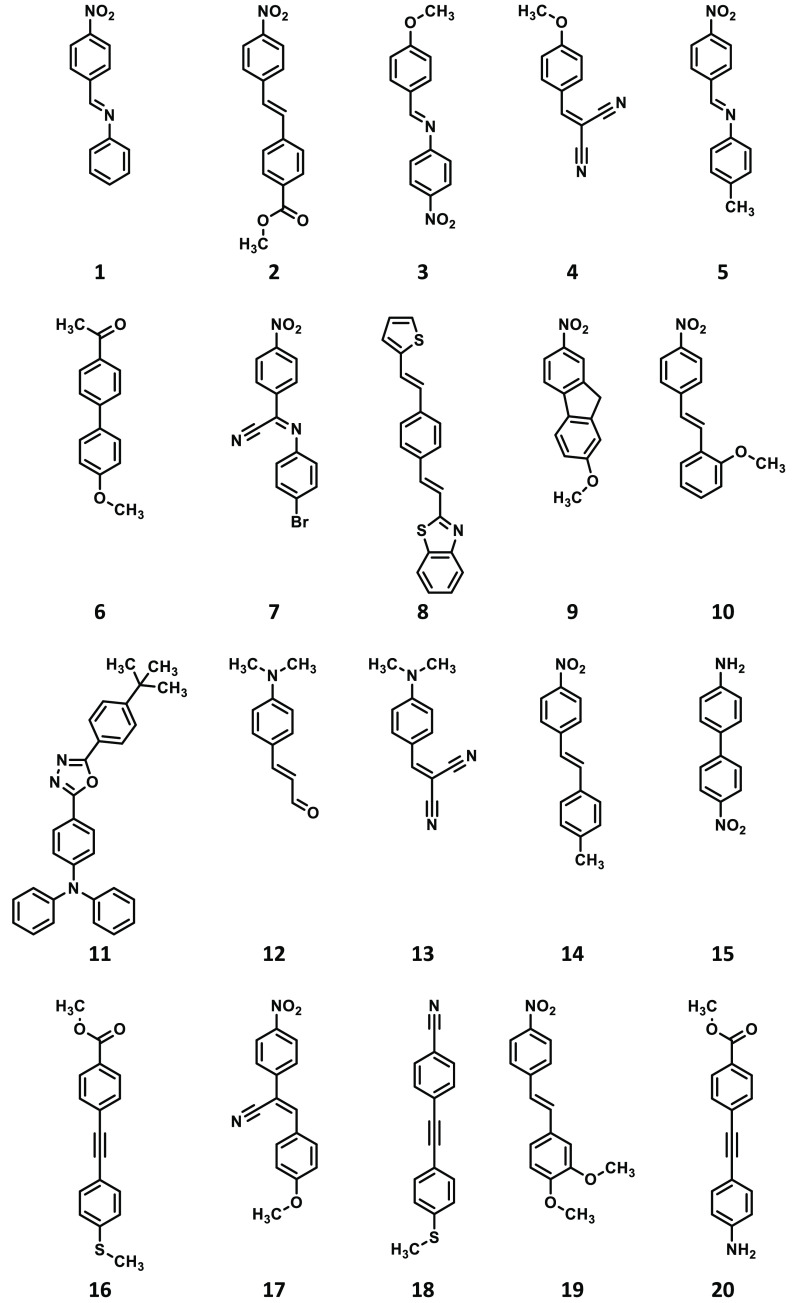
Structures of Compounds **1**–**20** Studied in the Present Work

**Scheme 2:**
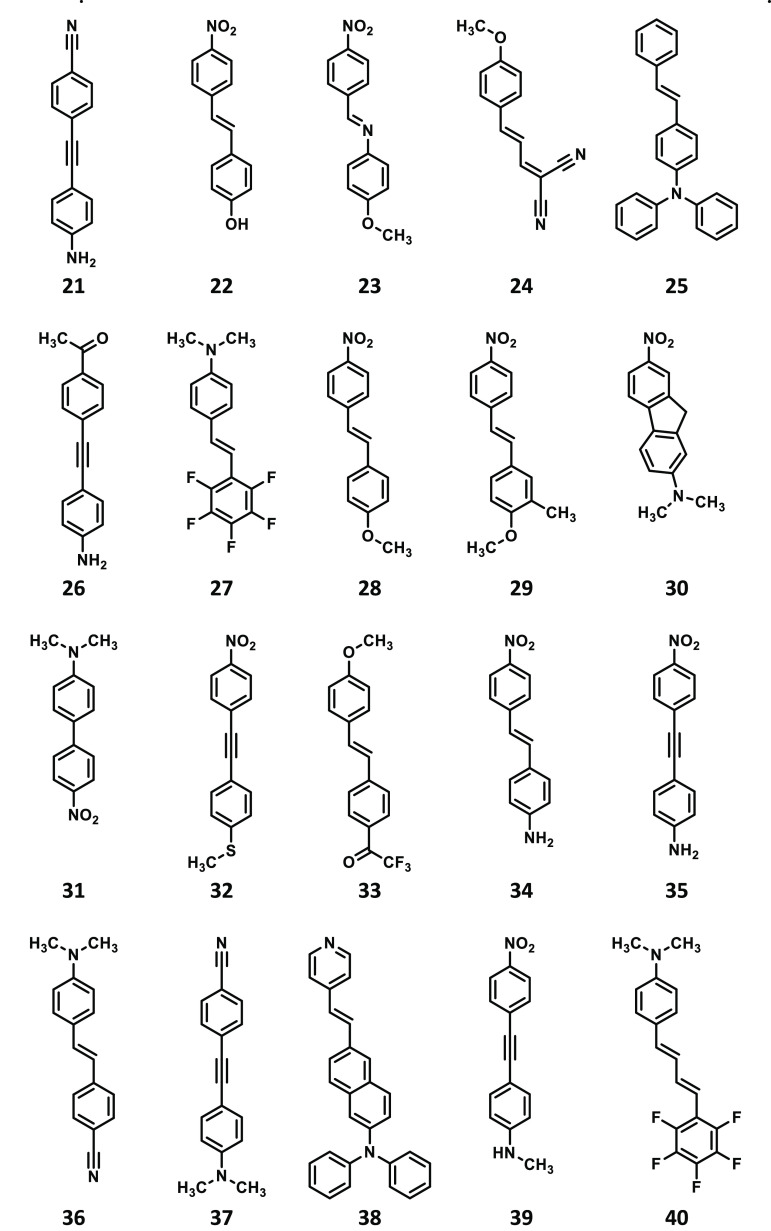
Structures of Compounds **21**–**40** Studied in the Present Work

**Scheme 3:**
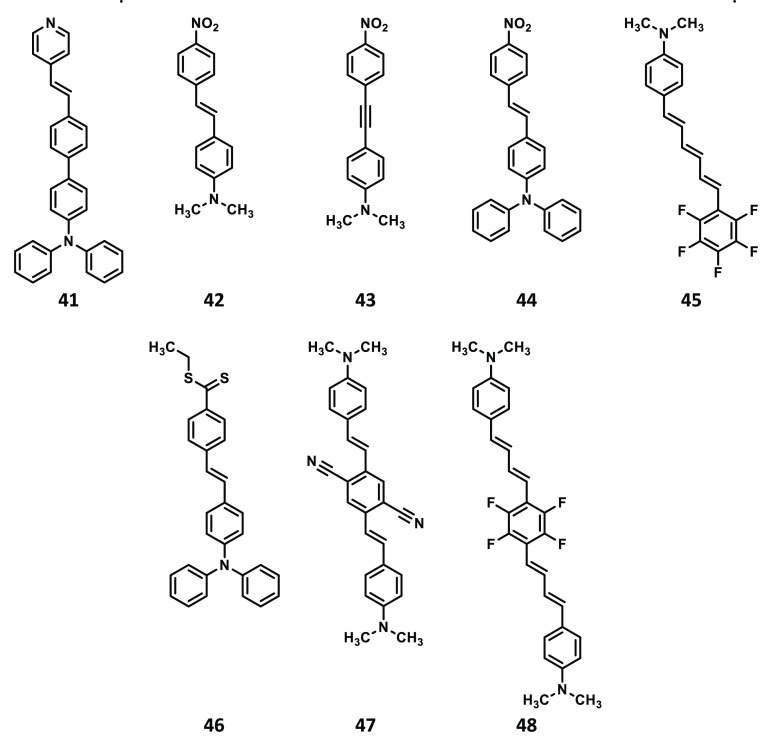
Structures of Compounds **41**–**48** Studied in the Present Work

Taken together, the developments summarized above demonstrate the necessity to perform an extensive comparative study to validate the performance of range-separated functionals in predicting 2PA spectra. The present contribution aims at filling this gap. More specifically, the goal of the present work is to evaluate the performance of selected DFAs, including OT-RSFs, in predicting electronic 2PA transition strengths for the series of 48 organic molecules shown in Schemes 1–3. The chosen compounds are examples of donor–acceptor (D–A) and donor–acceptor–donor (D–A–D) systems (covering a wide range of donor/acceptor strengths for the substituents) whose third-order (resonant and nonresonant) properties have been thoroughly studied experimentally.^81−83^ The choice of these systems stems from the fact that D–A and D–A–D architectures are widely employed in the design of two-photon active materials. Especially the D–A motif leads to bright low-lying states with large dipole moment changes upon excitations (intramolecular charge-transfer transitions; see Figures S1 and S2): features highly beneficial for large two-photon-absorption cross sections. As will be demonstrated in the remainder of this work, many of the chosen D–A and D–A–D compounds exhibit significant two-photon strengths, thus making them suitable candidates for this study. No studies have to date reported the performance of OT-RSFs for determining the 2PA cross sections, further justifying the need for assessing these types of functionals. In order to achieve these goals, the resolution-of-identity CC2 model^84^ will be used as reference method and a thorough analysis of electronic-structure parameters will be performed to pinpoint the origins of the differences in performance for various DFAs.

The remainder of the paper is organized as follows. In section 2, we briefly summarize the theoretical foundations for 2PA and the generalized few-state models that we will use to analyze the results. In section 3 we summarize the computational details. Subsequently, we present and discuss our findings in section 4. Finally, in section 5 we give some concluding remarks.

## 2 Theory

### 2.1 Description of 2PA in Hermitian and Non-Hermitian Theories

Within the framework of Hermitian (H) and non-Hermitian (NH) theories, the rotationally averaged two-photon transition strengths for the |0⟩ → |*J*⟩ transition, in the case of a single beam of linearly polarized monochromatic light, are given by^52^
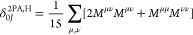
1a
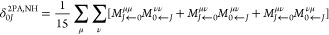
1bwith
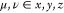


*M*_*J*←0_^*μν*^ and *M*_0←*J*_^*μν*^ denote the *μν*th component of the right and left second-order transition moments, respectively, in the non-Hermitian description. In the case of the Hermitian counterpart, there is no difference between the left and right transition moments; hence it is represented by *M*^*μν*^, without any subscript. The sum-over-states expressions for the transition moments are given by
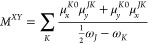
2a
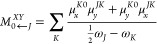
2b
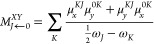
2cwhere ω_*K*_ represents the excitation energy for the |0⟩ → |*K*⟩ transition and μ_*x*_^*KL*^ = ⟨*K*|*X*|*L*⟩ in eqs 2a–2c is the *x*-component of the first-order transition dipole moment for the |*K*⟩ → |*L*⟩ transition. The superscripts on μ distinguish the right (*L*0) and left (0*L*) first-order transition moments.

### 2.2 Generalized Few-State Model (GFSM) from Hermitian and Non-Hermitian Theories

The expressions for δ_0*J*_^2PA^ in eqs 1a and 1b do not reflect directly the effect of dipole orientation on 2PA. This can be made more explicit by inserting eqs 2a–2c into eqs 1a and 1b and separating the magnitude of the transition moments from the angle (orientation) terms, thus leading to the GFSM expressions.^63,85^ The final equation for the 2PA strength for a GFSM for the Hermitian theories is^85^
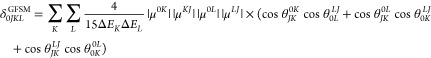
3and that for the non-Hermitian theories is^63^
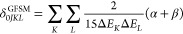
4with
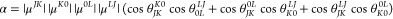

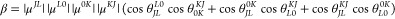


In the above expressions,  and the term θ_*PQ*_^*RS*^ represents the angle between the transition dipole moment vectors μ_*PQ*_ and μ_*RS*_.

Expressions for different few-state models can be derived from eqs 3 and 4 by choosing a given number of intermediate states *K* and *L*. For a two-state model (2SM), as used in this work, *K* and *L* can be either the ground state 0 or the final excited state *J*. The sum over *K* and *L* thus reduces to four terms: δ_0*J*00_, δ_0*J*0*J*_ ≡ δ_0*JJ*0_, and δ_0*JJJ*_, for which we will use the short-hand notation δ_00_, δ_0*J*_ ≡ δ_*J*0_, and δ_*JJ*_, respectively, in this work. Explicit expressions for Hermitian theories are then given by
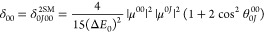
5
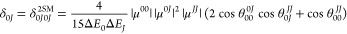
6
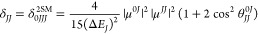
7

In the context of truncated sum-over-state (SOS) approaches, it should be mentioned that wave function based correlated methods introduce errors in the description of valence states located above the ionization threshold. Hence, considering a large number of the excited states in the SOS series can cause inaccuracies.^86^

## 3 Computational Details

The geometries of all 48 compounds shown in Schemes 1–3 were optimized in the gas phase by using the B3LYP functional^87^ and the cc-pVTZ basis set^88^ employing the Gaussian 16 program.^89^ The optimized ground-state geometries were confirmed to be minima by evaluation of the Hessian. Gas-phase electronic structure calculations were performed with the GAMESS US program^90^ at the optimized geometries to determine the one- and two-photon-absorption spectral parameters.^91^ The palette of exchange–correlation functionals used consisted of semilocal functionals (BLYP^92,93^ and PBE^94^), global hybrids (B3LYP^87^ and PBE0^95,96^), and range-separated hybrids (CAM-B3LYP^54^ and LC-BLYP^55^). The value of the range-separation parameter ω was set to 0.33 in the latter two functionals. The aug-cc-pVDZ basis set was employed in all DFA-based calculations. In addition, RI-CC2 calculations were performed with the use of the TURBOMOLE program.^84,97^ In these calculations, the aug-cc-pVDZ basis set^88^ and the corresponding recommended auxiliary basis set^98^ were used to determine the electronic structure and 2PA cross sections. The RI-CC2-based results will serve as reference when evaluating the performance of the DFAs. In a recent study, we have demonstrated that this method reproduces experimental trends in 2PA spectra for a family of donor–acceptor-substituted organic dyes.^63^ Moreover, higher-level calculations have verified the satisfactory performance of the CC2 method in predicting 2PA cross sections of organic chromophores.^62,99^

Additionally, the optimal tuning of the LC-BLYP functional was performed, resulting in a functional labeled as OT-LC-BLYP. For all but three of the systems studied, we have used the constraint of providing the closest agreement to Janak’s theorem for both the neutral, IP^*N*^, and anionic, IP^*N*+1^, species:^69,70^
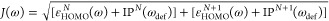
8with the *J*(ω) function being minimized with respect to the attenuation parameter ω. *N* is here the number of electrons in the neutral molecule, and ε_HOMO_^*N*^ is the energy of the highest occupied molecular orbital. The optimal value of the parameter, ω_opt_, was located by using the golden-section search.^100^ Three of the molecules had negative IP^*N*+1^ values at the LC-BLYP level of theory, and for these systems we only used the constraint for the IP of the neutral system.

In this study, we have kept the values of IP^*N*^(ω_def_) and IP^*N*+1^(ω_def_) frozen during the minimization of the *J*(ω) function. They were obtained with the default value of the attenuation parameter in LC-BLYP, as used in the Gaussian 16 program,^89^ namely, ω_def_ = 0.47 bohr^–1^. The use of fixed values for IP^*N*^(ω_def_) and IP^*N*+1^(ω_def_) has a minor impact on the value of the optimized ω_opt_. Optimization with the unfrozen IPs yields ω_opt_ up to 0.04 bohr^–1^ lower, but this has been shown to only have minor consequences for the computed first hyperpolarizabilites (not exceeding 20%).^80^

## 4 Results and Discussion

We will start with an analysis of the two-photon-absorption strengths computed with response theory using both the reference RI-CC2 method and different DFAs (BLYP, B3LYP, PBE, PBE0, (OT)-LC-BLYP, and CAM-B3LYP) for the whole set of 48 molecules. This is followed by a more detailed analysis using a two-state model (2SM) on a subset of these molecules. We then discuss in greater detail the results obtained using OT-LC-BLYP, before we end by a comparison with some more heavily parametrized DFAs. Note that the compounds in Schemes 1–3 have been ordered by increasing values of δ^2PA^ as obtained at the RI-CC2/aug-cc-pVDZ level of theory.

### 4.1 Analysis of 2PA Strengths

**Figure 1:**
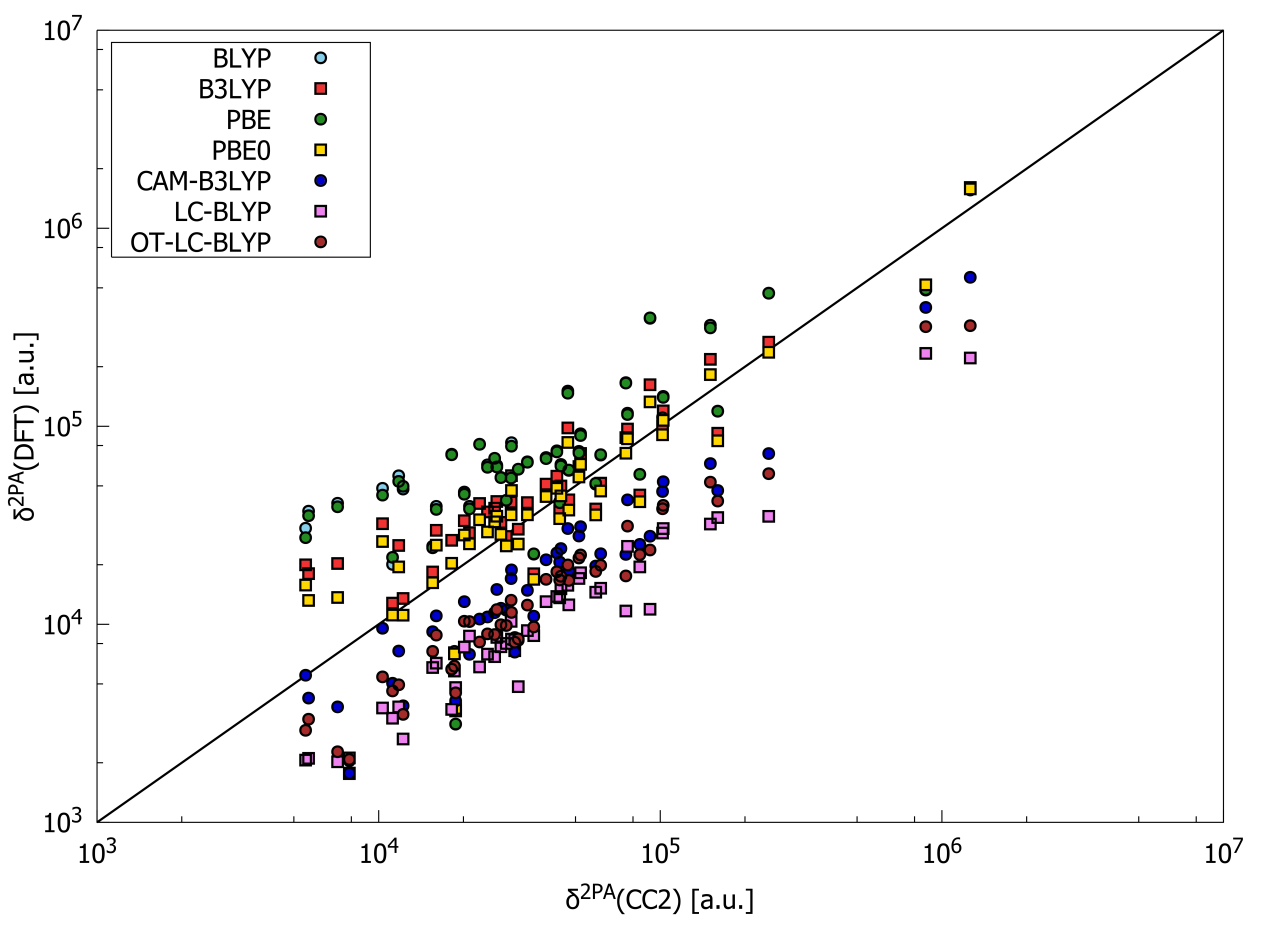
Comparison of two-photon-absorption strengths computed using RI-CC2 method and density functional approximations (double logarithmic scale is used with base 10). See Table 1 for the linear regression data.

**Figure 2:**
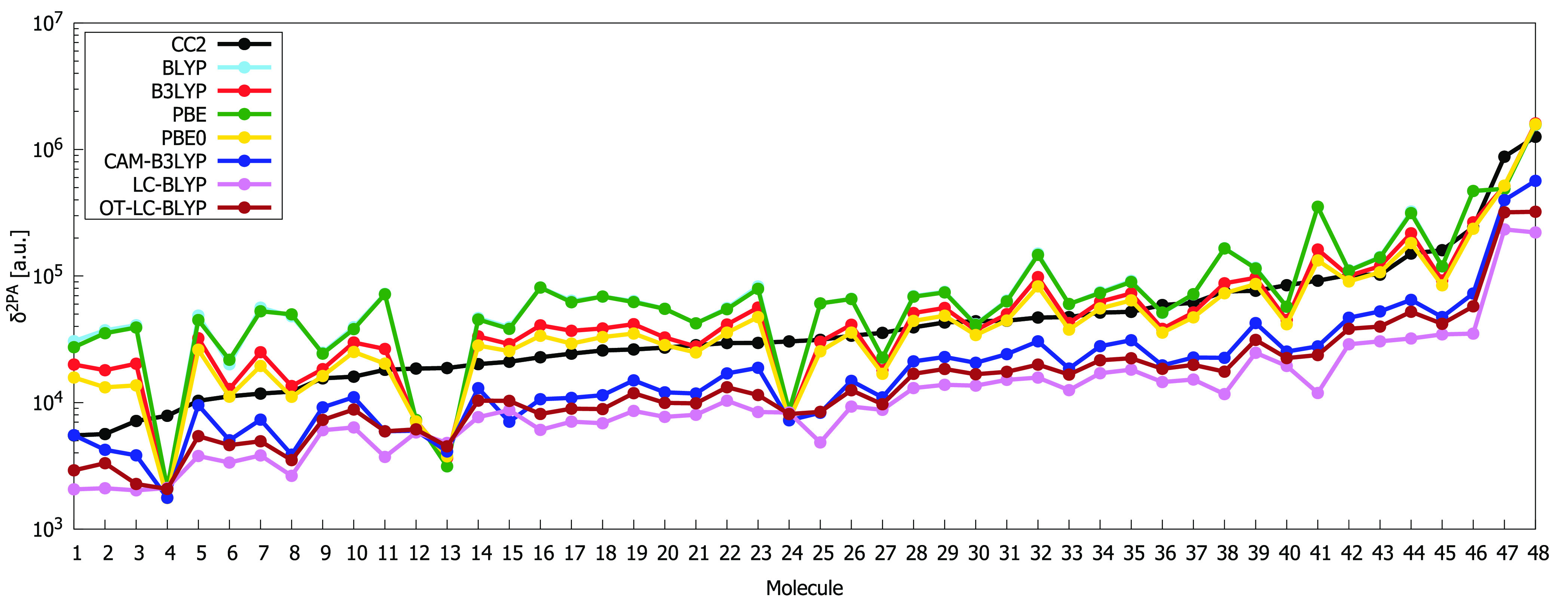
Two-photon-absorption strengths computed using RI-CC2 method and density functional approximations (logarithmic scale is used with base 10).

Figures 1 and 2 compare δ^2PA^ values obtained by using RI-CC2 and different DFAs (see also Figures S3–S16). Relative 2PA strengths when going from one molecule to the next in the series are presented in Figures S14 and S15. As can be seen, PBE0 provides results that on average are closest to those obtained by RI-CC2. Nevertheless, even the PBE0 values are far from satisfactory and many molecules present unsystematic errors relative to the RI-CC2 reference. Indeed, the differences between 2PA strengths as predicted by RI-CC2 and PBE0 range from a few thousand atomic units to over 300 000 au (see Tables S1 and S2). On the basis of the analysis of the data shown in Figures 1 and 2 and collected in Tables S1 and S2, it is clear that the performances of all DFAs used to calculate δ^2PA^ for the selected set of compounds are in general poor. Interestingly, all range-separated functionals (RSFs), i.e., CAM-B3LYP, LC-BLYP, and OT-LC-BLYP, underestimate δ^2PA^ for all molecules, the only exception being CAM-B3LYP for molecule **1**. The remaining four functionals (BLYP, B3LYP, PBE, and PBE0) both over- and underestimate 2PA strengths with respect to RI-CC2. Among the DFAs considered, the pure GGA functionals BLYP and PBE provide in most cases the largest values of δ^2PA^, whereas the smallest values are in most cases obtained with LC-BLYP.

When passing from molecule **1** to molecule **48**, the results obtained by all DFAs demonstrate their nonmonotonic behavior (see Figure 2). The qualitative trends predicted by BLYP and PBE as well as by B3LYP and PBE0 are in each case very similar. Quantitatively, BLYP and PBE provide very similar 2PA strengths. The 2PA strengths provided by B3LYP are somewhat larger than those obtained with PBE0, with molecule **47** as the only exception. It is clear from Figure 2 that there are some significant irregularities in the predicted 2PA strengths for the DFAs. In particular, the performances of BLYP, B3LYP, PBE and PBE0 are poor for molecules with a terminal cyano group (molecules **4**, **13**, **24**).

In Table 1, we collect the data for the linear regression plots between the 2PA strengths calculated with RI-CC2 and each DFA. Each type of DFA (pure GGA, hybrid, or RSF) behaves differently, suggesting that the amount of Hartree–Fock exchange plays an essential role in the accuracy of the different DFAs. The best agreement with RI-CC2 2PA strengths is obtained by using hybrid functionals (which show a slope close to 1 and a small intercept), whereas all RSFs severely underestimate 2PA strengths by approximately 45, 20, and 29% for CAM-B3LYP, LC-BLYP, and OT-LC-BLYP, respectively. Even though RSFs clearly understate the absolute 2PA strengths, they mostly predict the same change in relative 2PA strength as RI-CC2 (notice the relatively high Pearson coefficient, especially in the case of CAM-B3LYP).

**Table 1:** Data from the Linear Regression Plots between the 2PA Strengths Calculated with RI-CC2 and Each DFA^a^

DFA	*m*	*a*	*r*^2^
BLYP	1.01	31 732	0.85
PBE	1.02	30 336	0.85
B3LYP	1.05	2162	0.90
PBE0	1.04	4195	0.91
CAM-B3LYP	0.45	–1009	0.99
LC-BLYP	0.20	2824	0.95
OT-LC-BLYP	0.29	2852	0.96

a *m* is the slope, *a* is the intercept, and *r*^2^ is the Pearson coefficient. Units (*a*) are au. See also Figure 1.

### 4.2 Few-State-Model Analysis

**Figure 3:**
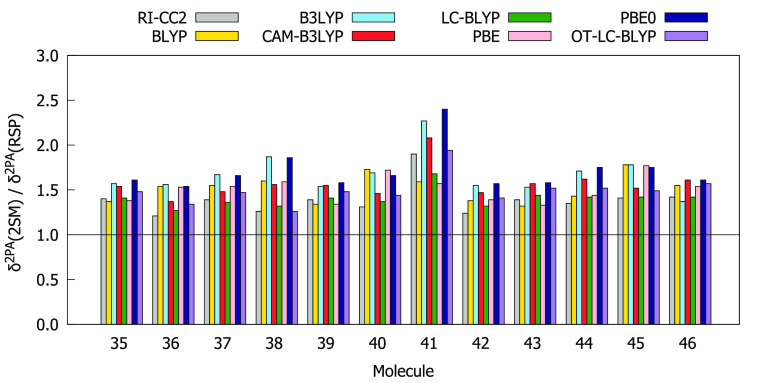
Ratios between two-photon-absorption strengths δ^2PA^ computed with two-state model (2SM) and with response theory (RSP) using the aug-cc-pVDZ basis set.

To gain insight into the reasons for the abilities of different DFAs to predict δ^2PA^ values of the studied molecules, we employed the generalized few-state model^63,85^ described in section 2.2. We will focus on molecules **35**–**46**, which is a subset composed of prototypical push–pull dipolar compounds with large 2PA strengths. For this subset, we compared δ^2PA^ values obtained from a 2SM for the lowest bright ππ* excited state in these systems (see Table S3). The 2SM has in general been shown to work well for dipolar structures,^21^ but we will nevertheless compare the 2SM-based 2PA strengths with the results obtained using response (RSP) theory. We present the ratios between δ^2PA^’s calculated with the 2SM and response theory in Figure 3.

The values of δ^2PA^(2SM) are for all molecules higher than the values of δ^2PA^(RSP). This overestimation is however less than a factor of 2 for all molecules, except molecule **41** for B3LYP, PBE0, and CAM-B3LYP. With the exception of molecule **41**, RI-CC2 and LC-BLYP provide 2SM results that are most consistent with the response theory values (δ^2PA^(2SM)/δ^2PA^(RSP) < 1.5). The remaining functionals are less systematic, as there are several instances where the ratio between δ^2PA^(2SM) and δ^2PA^(RSP) exceeds 1.5. A three-state model does not give better agreement with the response theory results, as can be seen from Figure S16. For the above reasons, the 2SM was selected for further analysis for all structures of the chosen subset.

We will now proceed with an analysis of all terms contributing to the two-photon-absorption strength within a two-state approximation, i.e., δ_00_, δ_0*J*_, δ_*J*0_, and δ_*JJ*_, respectively; see section 2.2 for definitions. Table S5 collects the percentage contributions of these terms to the δ^2PA^(2SM). Note that δ_0*J*_ = δ_*J*0_ and hence only their sum is presented in Table S5 as one contribution, δ_0*J*+*J*0_. In all cases, δ_0*J*_ + δ_*J*0_ is negative whereas δ_00_ and δ_*JJ*_ are positive, with the smallest contribution coming from δ_00_. For all molecules studied, the absolute values of δ_*JJ*_ prevail over the other two terms for RI-CC2 as well as for the BLYP, B3LYP, and PBE functionals. For the CAM-B3LYP, LC-BLYP, PBE0, and OT-LC-BLYP functionals, either δ_0*J*_ + δ_*J*0_ or δ_*JJ*_ has the largest absolute value depending on the molecule. Therefore, a proper description of these two terms is important for a correct determination of δ^2PA^(2SM) and hence also for δ^2PA^(RSP).

**Figure 4:**
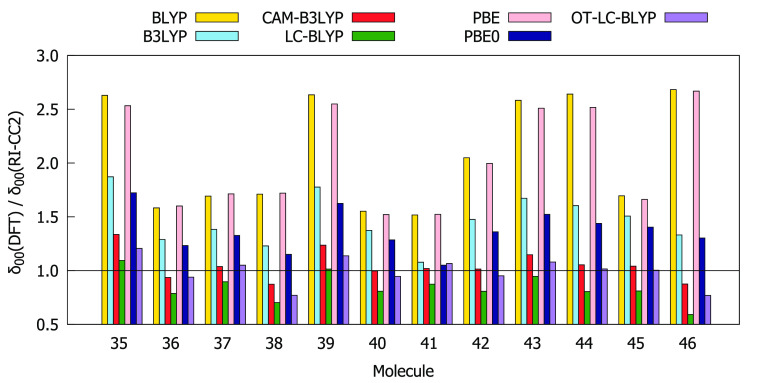
Ratios between δ_00_ computed at DFT and RI-CC2 levels using the aug-cc-pVDZ basis set.

**Figure 5:**
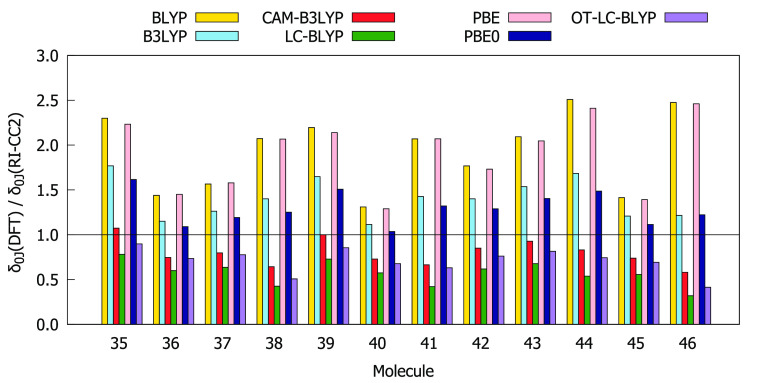
Ratios between δ_0*J*_ computed at DFT and RI-CC2 levels using the aug-cc-pVDZ basis set.

**Figure 6:**
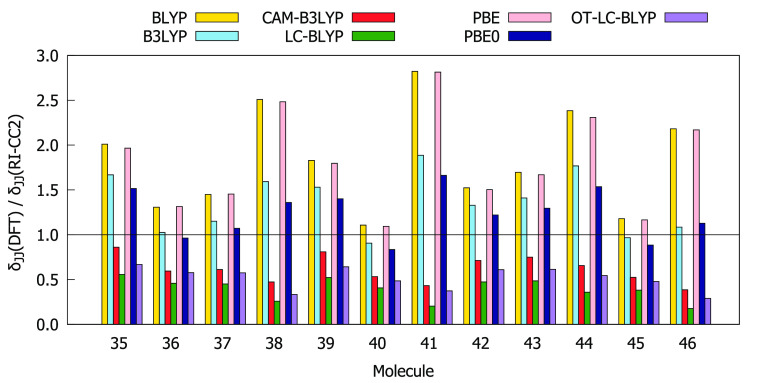
Ratios between δ_*JJ*_ computed at DFT and RI-CC2 levels using the aug-cc-pVDZ basis set.

Comparisons of the values for δ_00_, δ_0*J*_ + δ_*J*0_, and δ_*JJ*_ obtained using the RI-CC2 method and different DFAs are given in Figures 4–6. The BLYP, PBE, B3LYP, and PBE0 functionals overestimate both δ_00_ and δ_0*J*_ compared to the RI-CC2 reference for the entire subset of molecules. In many cases, this overestimation exceeds a factor of 1.5. For all molecules, BLYP and PBE overestimate δ_*JJ*_ whereas CAM-B3LYP, LC-BLYP, and OT-LC-BLYP underestimate δ_*JJ*_. BLYP and PBE always provide the largest values of δ_00_ and δ_0*J*_ as well as of δ_*JJ*_, whereas LC-BLYP always provides the lowest values. In order to select functionals that work best for describing all three contributions to δ^2PA^(2SM), we have calculated the absolute values of deviation of the ratios between the DFT and RI-CC2 results from 1, that is, |1 – *A*(DFT)/*A*(CC2)|, where *A* is δ_00_, δ_0*J*_, or δ_*JJ*_. The results are collected in Table S13. The analysis shows that for molecules **35**–**46** the values of δ_00_, δ_0*J*_, and δ_*JJ*_ provided by OT-LC-BLYP, CAM-B3LYP, and PBE0, respectively, are in the best agreement with RI-CC2 results.

**Figure 7:**
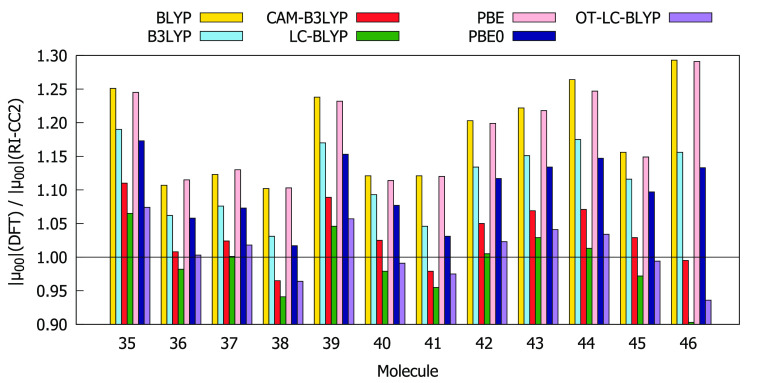
Ratios between ground-state dipole moment (|μ_00_|) values computed at DFT and RI-CC2 levels using the aug-cc-pVDZ basis set.

**Figure 8:**
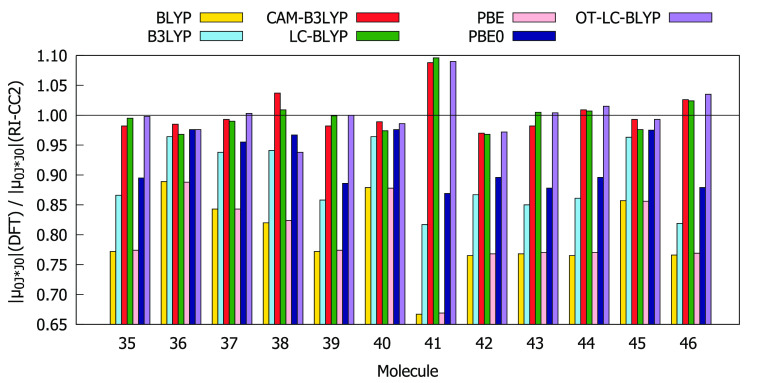
Ratios between transition moment (|μ_0*J*·*J*0_| = ) values computed at DFT and RI-CC2 levels using the aug-cc-pVDZ basis set.

**Figure 9:**
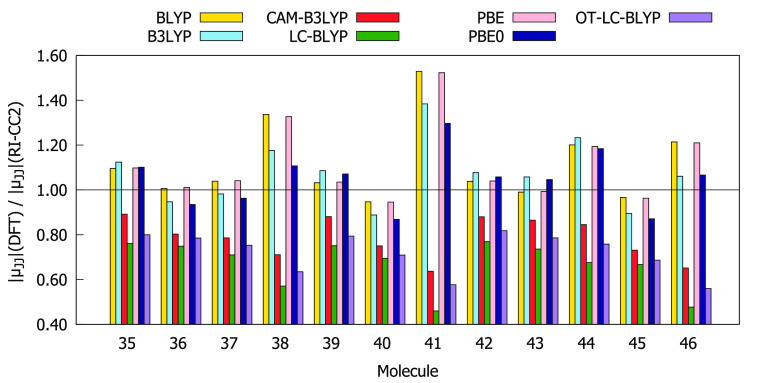
Ratios between excited-state dipole moment (|μ_*JJ*_|) values computed at DFT and RI-CC2 levels using the aug-cc-pVDZ basis set.

**Figure 10:**
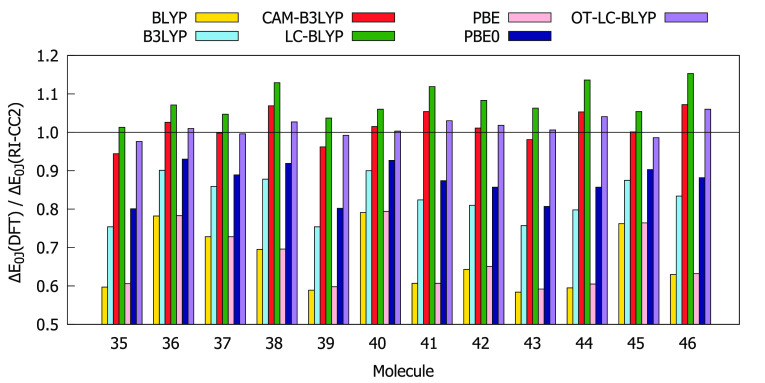
Ratios between excitation energies (Δ*E*_0*J*_) computed at DFT and RI-CC2 levels using the aug-cc-pVDZ basis set.

We take a closer look at the spectroscopic parameters of molecules **35**–**46**, i.e., the ground-state dipole moment (|μ_00_|), the transition moment (due to the fact that right and left transition moments differ for non-Hermitian theories, we will discuss their product)
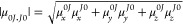
the excited-state dipole moment (|μ_*JJ*_|), and the excitation energy (Δ*E*_0*J*_). As can be seen from Figures 7–10, the values of |μ_00_|, |μ_0*J*·*J*0_|, and Δ*E*_0*J*_ obtained using all exchange–correlation functionals are in good agreement with the RI-CC2 data, as the ratios between DFT and RI-CC2 results range from 0.5 to 1.3. Slightly worse behavior was observed for |μ_*JJ*_| (0.4 < |μ_*JJ*_|(DFT)/|μ_*JJ*_|(CC2) < 1.6). BLYP, PBE, B3LYP, and PBE0 overestimate |μ_00_| and underestimate |μ_0*J*·*J*0_| and Δ*E*_0*J*_ for compounds **35**–**46**. The behavior of CAM-B3LYP, LC-BLYP, and OT-LC-BLYP in the descriptions of |μ_00_| and |μ_0*J*·*J*0_| varies depending on the molecule in question. In contrast, the excited-state dipole moments provided by CAM-B3LYP, LC-BLYP, and OT-LC-BLYP are always underestimated, whereas the remaining functionals do not display a systematic behavior. Note also that, for all molecules, BLYP and PBE (LC-BLYP) predict values for |μ_00_|, |μ_0*J*·*J*0_|, and Δ*E*_0*J*_ (|μ_*JJ*_|) that differ the most from RI-CC2 reference values. The values of |μ_00_| and Δ*E*_0*J*_ provided by OT-LC-BLYP for compounds **35**–**46** are on average closest to the reference data (see Table S13). The same applies to LC-BLYP and |μ_0*J*·*J*0_| as well as to PBE0 and |μ_*JJ*_|.

### 4.3 Analysis of Optimally Tuned LC-BLYP Results

We will now consider in greater detail the performance of the optimally tuned LC-BLYP (OT-LC-BLYP). As can be seen from Figure 2, the OT-LC-BLYP functional gives slightly better agreement with RI-CC2 for δ^2PA^(RSP) compared to LC-BLYP for all molecules except **4**, **13**, and **24**. Nevertheless, the values provided by OT-LC-BLYP are significantly underestimated compared to the RI-CC2 results. The 2PA strengths calculated using a 2SM and OT-LC-BLYP for most of molecules from **35** to **46**, except **41**, are in good agreement with the corresponding RSP results, as the ratios between δ^2PA^(2SM) and δ^2PA^(RSP) range from 1.26 to 1.57 (Figure 3). OT-LC-BLYP always predicts the values of δ_00_, δ_0*J*_, and δ_*JJ*_ to be higher than the ones obtained with LC-BLYP, in most cases giving improved performance compared to LC-BLYP. Moreover, considering only the two dominating contributions to δ^2PA^(2SM), δ_0*J*_ and δ_*JJ*_, it is clear that OT-LC-BLYP works best, since it shows the best systematic behavior in predicting δ_0*J*_ and δ_*JJ*_ among all DFAs for molecules **35**–**46** (i.e., δ_0*J*_ and δ_*JJ*_ of all these compounds are always underestimated by OT-LC-BLYP) and provides values that in most cases are in fairly good agreement with RI-CC2 data. These two facts give OT-LC-BLYP an advantage over other DFAs in studies of δ^2PA^(2SM), since the systematic behavior of OT-LC-BLYP allows error cancellations to be minimized. This also applies to the calculated response theory values of δ^2PA^, as the two-state model is a reliable approximation to the RSP values for most of the molecules considered in the case of OT-LC-BLYP. Note that, according to eq 3, |μ_00_|, |μ_*JJ*_|, and Δ*E*_0*J*_ directly contribute to δ_0*J*_ and δ_*JJ*_. We can therefore pinpoint the underestimated excited-state dipole moment as the main source of the underestimation of δ_0*J*_ and δ_*JJ*_ and hence also δ^2PA^(RSP) by OT-LC-BLYP. This is in agreement with previous observations.^63^ A similar analysis and conclusions can be made for the other RSFs. In fact, a recent benchmark study of ground- and excited-state dipole moments of organic molecules by Jacquemin demonstrates that RSFs suffer from underestimated values of dipole moment differences corresponding to ππ* transitions.^101^ For a systematic analysis of the performance of DFAs in predicting ground- and excited-state dipole moments of small and medium-sized molecules, we refer to extensive benchmark works.^102−104^

### 4.4 Analysis of Heavily Parametrized Functionals

As highlighted above, RSFs yield systematically underestimated values of two-photon transition strengths in comparison with RI-CC2 reference values and this observation can be linked to large errors in excited-state dipole moments. In order to explore this matter further, we selected two highly parametrized Minnesota functionals (M06-2X^105^ and MN15^106^) and ωB97X-D.^107^ Since calculations of 2PA strengths using these functionals are not possible using publicly available software releases, we have estimated the 2PA strengths using the two-state approximation, as we have already shown that this model captures the main features of the 2PA strengths. The necessary excited-stated dipole moments were calculated using the Z-vector method^108,109^ (as implemented in the Gaussian 16 program^89^).

1,2-Diphenylethene and 1,2-diphenylethyne derivatives (**35**–**37**, **39**, **42**, **43**) were selected for this analysis as they represent typical push–pull systems with significant charge transfer in the lowest-lying electronic singlet excited state. A summary of the analysis is shown in Table 2. Three key conclusions can be drawn from this analysis: (1) DFAs having the largest errors in the 2PA strengths also have the largest errors in the excited-state dipole moments compared to RI-CC2; (2) the range-separated ωB97X-D functional does not provide any systematic improvement over other RSFs; (3) for the studied subset of molecules, we notice a systematic underestimation of the 2PA strengths by the Minnesota functionals, but with much smaller errors relative to RI-CC2 than the RSFs, with MN15 being slightly more accurate than its predecessor, M06-2X.

**Table 2:** Relative Errors in δ^2PA^ within the Two-State Approximation and in Dipole Moment in the S_1_ Excited State with Respect to CC2 Reference^a^

	B3LYP (20%)		PBE0 (25%)		MN15 (44%)		M06-2X (54%)		CAM-B3LYP (65%)		ωB97X-D (100%)	
molecule	δ^2PA^	|μ|	δ^2PA^	|μ|	δ^2PA^	|μ|	δ^2PA^	|μ|	δ^2PA^	|μ|	δ^2PA^	|μ|
**35**	**56.69**	**12.42**	**41.37**	**10.11**	–6.96	–2.49	–25.93	–6.95	–34.38	–10.76	–54.63	–19.19
**36**	–16.46	–5.30	–22.86	–6.49	**–49.24**	**–15.80**	**–53.82**	**–16.27**	**–62.22**	**–19.67**	**–69.55**	**–22.93**
**37**	1.22	–1.81	–7.99	–3.65	**–43.26**	**–15.77**	**–47.75**	**–16.08**	**–60.41**	**–21.40**	**–70.45**	**–26.21**
**39**	**40.71**	**8.62**	**43.03**	**7.06**	–12.99	–4.29	–29.37	–8.06	–37.93	–11.89	–57.48	–20.35
**42**	23.86	7.70	13.40	5.79	–25.03	–5.72	–40.74	–9.75	–45.42	–12.01	–61.50	–19.23
**43**	28.58	5.77	18.56	4.58	–19.22	–6.26	–33.83	–9.68	–42.10	–13.51	–60.66	–22.09

a Shown are the values for two-photon S_0_ → S_1_ transition. In parentheses are the percentages of Hartree–Fock exchange at long range of the DFA.

The best-performing DFAs for this set of molecules are found to be global hybrids with a variable percentage of Hartree–Fock exchange: B3LYP (20%), PBE0 (25%), and MN15 (44%). However, the trends are not systematic. Molecules **35** and **39** require a relatively large percentage of HF exchange (44%), whereas **36** and **37** require smaller amounts (19% or less). On the other hand, properties of molecules **42** and **43** are better reproduced by PBE0, which has 25% Hartree–Fock exchange. For none of these systems do the long-range corrected DFAs provide accurate 2PA strengths, and the larger the amount of HF exchange at long range, the worse the results. These conclusions are at odds with a recent benchmark study by Andruniów et al.,^66^ who found that long-range corrected DFAs (in particular, CAM-B3LYP) give the most accurate 2PA strengths for a set of eight chromophores. Since the current set of molecules includes donor–acceptor molecules with a large variation in 2PA strengths (ranging from 5000 to 10^6^ au), we conclude that the current set poses a greater challenge for current DFAs. In fact, no DFA provides accurate absolute 2PA strengths relative to RI-CC2 and is, at the same time, robust under two-state-model approximations.

Nayyar et al. studied two-photon-absorption spectra of a series of substituted symmetric oligophenylvinylenes using several DFAs: HSE06, B3LYP, M05, BMK, M05-2X, and CAM-B3LYP.^86^ These authors arrived at a somewhat different conclusion, i.e., only for some of the molecules studied did the CAM-B3LYP functional predict smaller values of the 2PA cross section than those predicted by B3LYP. Note that the set of oligophenylvinylenes was particularly problematic for the CAM-B3LYP functional, which predicted spurious multiple maxima in the absorption spectra, in contradiction to experimental data. It was demonstrated by Nayyar et al. that the B3LYP functional delivers the best estimates of 2PA cross sections with respect to the reference experimental data. The trends obtained in this work also suggest that B3LYP predicts values for 2PA cross sections closer to CC2, albeit due to cancellation of errors.

## 5 Summary and Conclusions

The two-photon-absorption strengths of a collection of medium-sized molecules have been studied using RI-CC2 and a selection of density functional approximations (DFAs). The molecules represent donor−π–acceptor architectures, and the lowest-energy bright ππ* state studied here indicates intramolecular charge-transfer character. Excitation to this electronic state is dominated by the one-electron HOMO → LUMO orbital transition. Given the type of excitation studied, we used the single-reference CC2 method, combined with the medium-sized aug-cc-pVDZ basis set, as the reference level to analyze performances of various DFAs.

Although the general wisdom has been that range-separated DFAs are best suited to study multiphoton absorption,^110,111^ the present study shows that they severely underestimate 2PA strengths due to a concomitant underestimation of the excited-state dipole moment. Despite providing abysmal absolute 2PA strengths, this class of DFAs shows the highest linear correlations with RI-CC2 results, as shown by the Pearson coefficient values. The best performing range-separated functionals are CAM-B3LYP and OT-LC-BLYP. On the other hand, global hybrids are more successful in reproducing the absolute values of 2PA strengths of many donor–acceptor molecules from the series but with worse Pearson coefficient values than in the case of range-separated functionals. Hence, we recommend the latter DFAs for “structure–property” studies across large series of dipolar compounds at the price of underestimated two-photon strengths.

The current set of molecules poses a challenge that no studied DFA successfully passed. None of the DFAs provided accurate absolute 2PA strengths for both response theory and a few-state-model approximation, although some of the highly parametrized functionals, e.g., MN15, showed systematic errors and predicted properties closer to reference ones. We are convinced this test set will prove valuable for developers of DFAs within the time-dependent density functional theory framework.

## Supporting Information Available

Results of time-dependent density functional calculations; numerical data corresponding to linear and quadratic response function calculations; results of two-state-model calculations (PDF)
