# Supplementary material for: Choosing Bad versus Worse: Predictions of Two-Photon-Absorption Strengths Based on Popular Density Functional Approximations
Source: J Chem Theory Comput. 2022 Jan 26;18(2):1046–60. doi: 10.1021/acs.jctc.1c01056 (PMC8830054; doi:10.1021/acs.jctc.1c01056)
Supplement: Supplementary file 1 — ct1c01056_si_001.pdf [file ct1c01056_si_001.pdf]

Supporting Information

Choosing Bad Versus Worse: Predictions of Two-Photon Absorption Strengths Based on Popular Density Functional Approximations

Marta Chołuj,[†] Md. Mehboob Alam,[*,‡] Maarten T. P. Beerepoot,[¶] Sebastian Sitkiewicz,[§,‖] Eduard Matito,[§,⊥] Kenneth Ruud,[*,¶] and Robert Zaleśny[*,†]

†*Faculty of Chemistry, Wrocław University of Science and Technology, Wyb. Wyspiańskiego 27, PL–50370 Wrocław, Poland*

‡*Department of Chemistry, Indian Institute of Technology Bhilai, Sejbahar, Raipur, Chhattisgarh – 492015, India*

¶*Hylleraas Centre for Quantum Molecular Sciences, Department of Chemistry, UiT The Arctic University of Norway, N-9037 Tromsø, Norway*

§*Donostia International Physics Center (DIPC), Manuel Lardizabal Ibilbidea 4, 20018 Donostia, Euskadi, Spain*

‖*Kimika Fakultatea, Euskal Herriko Unibertsitatea (UPV/EHU), 20080 Donostia, Euskadi (Spain)*

⊥*Ikerbasque Foundation for Science, Plaza Euskadi 5, 48009 Bilbao, Euskadi, Spain*

E-mail: mehboob@iitbhilai.ac.in; kenneth.ruud@uit.no; robert.zalesny@pwr.edu.pl

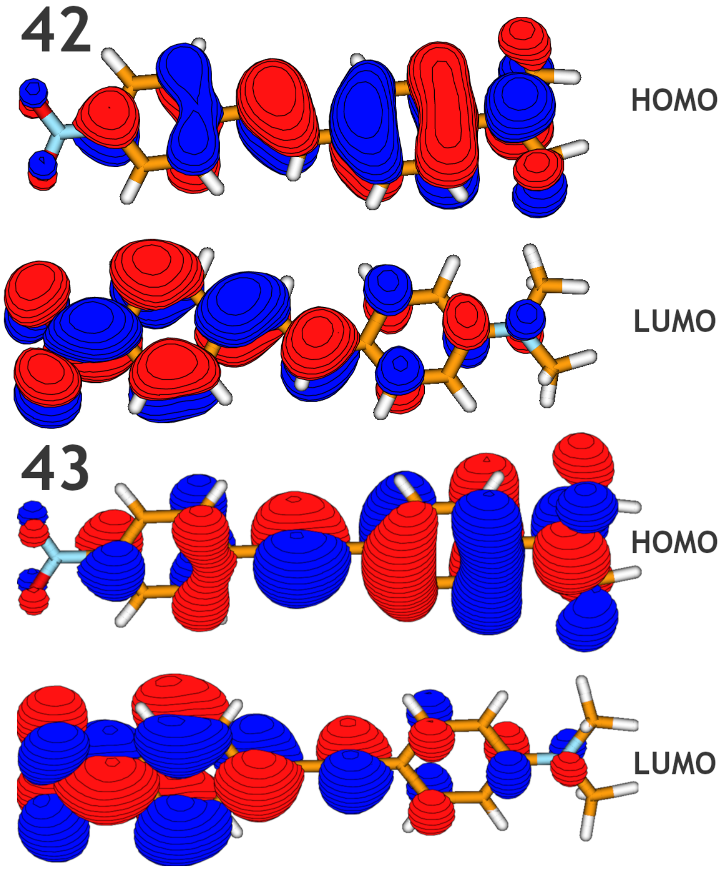

Figure S1: Frontier orbitals dominantly involved in $S_0 \rightarrow S_1$ electronic excitation (amplitude was set to 0.02).

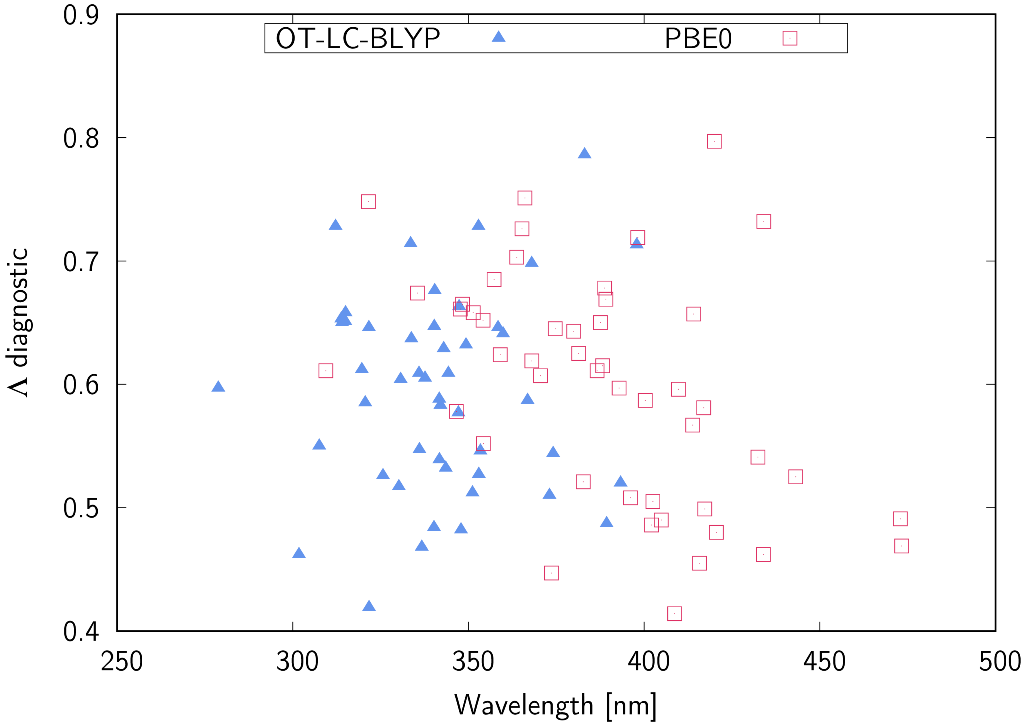

Figure S2: Λ diagnostic values computed using PBE0 and OT-LC-BLYP functionals.

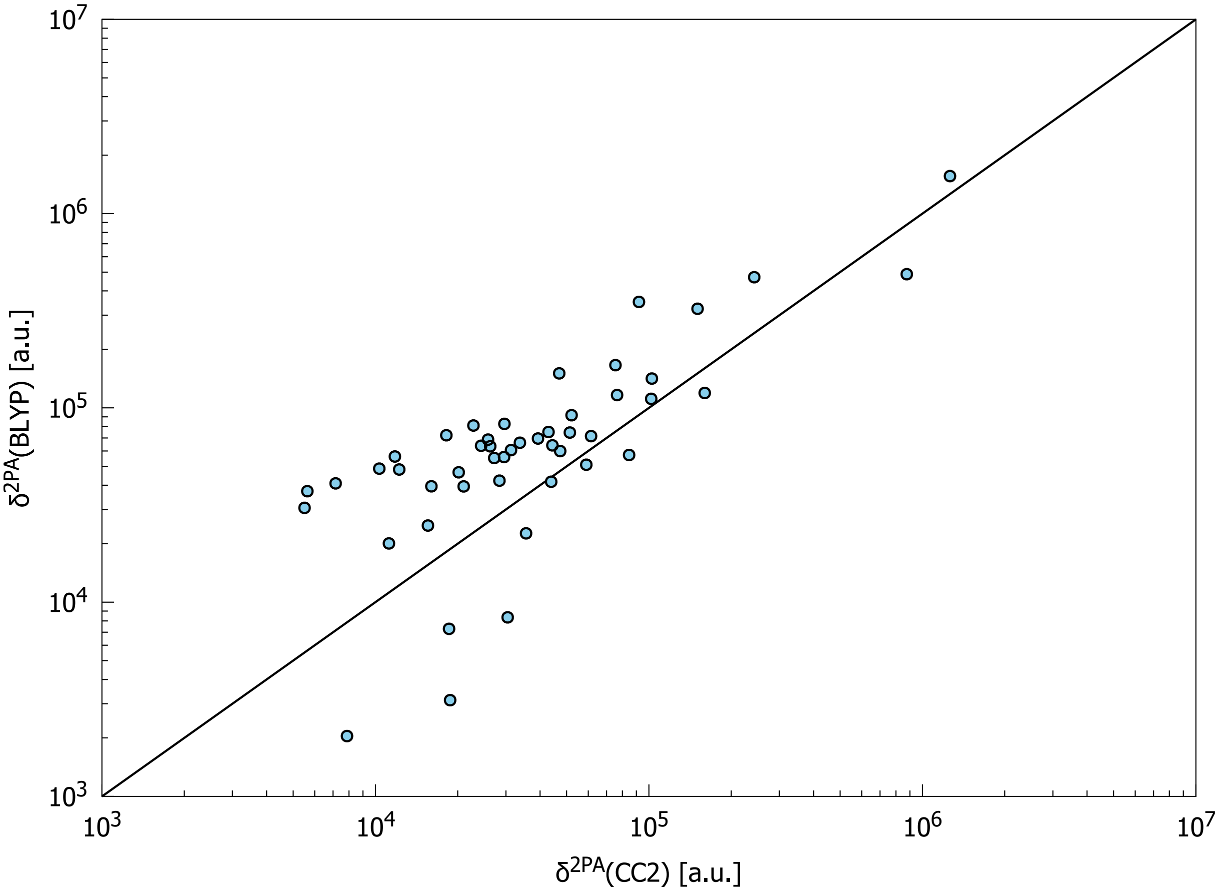

Figure S3: Comparison of two-photon absorption strengths computed using RI-CC2 method and BLYP functional (double logarithmic scale is used with base 10).

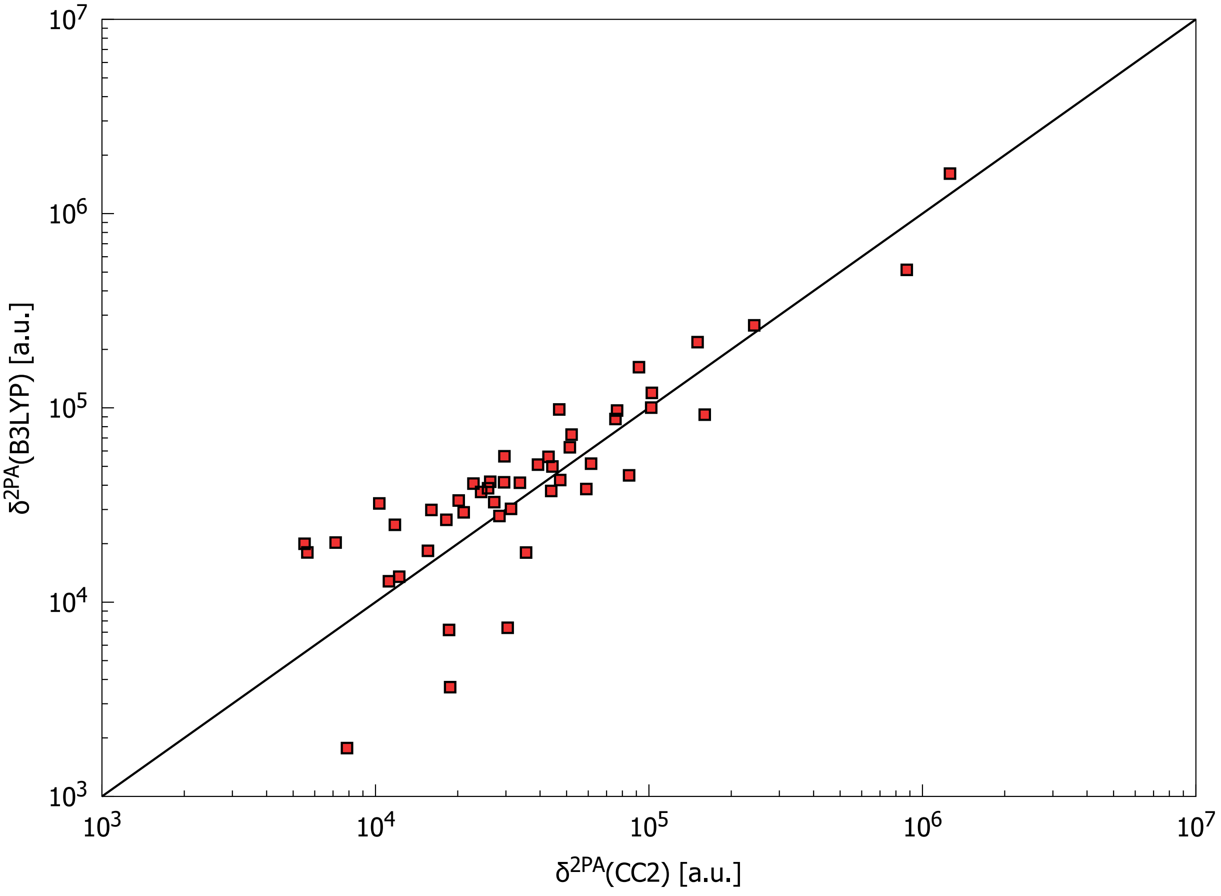

Figure S4: Comparison of two-photon absorption strengths computed using RI-CC2 method and B3LYP functional (double logarithmic scale is used with base 10).

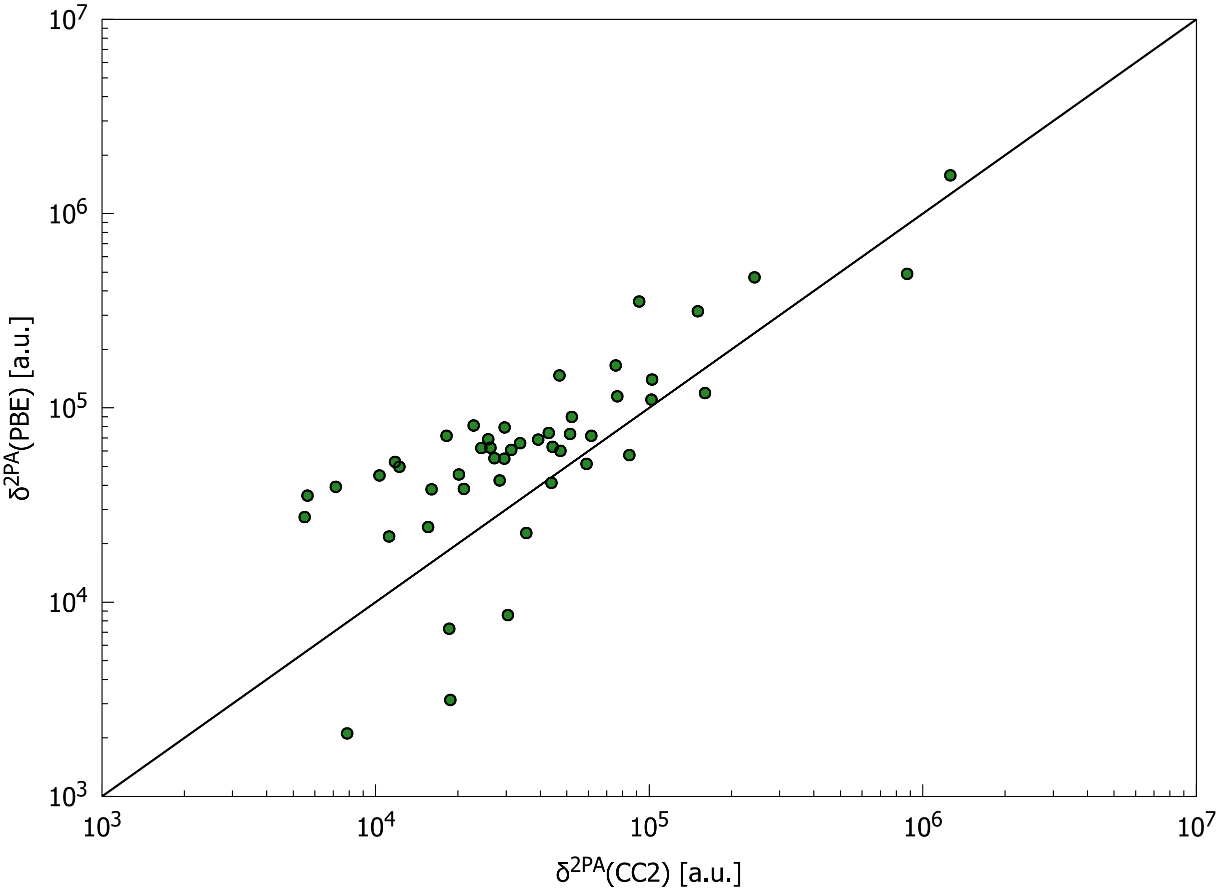

Figure S5: Comparison of two-photon absorption strengths computed using RI-CC2 method and PBE functional (double logarithmic scale is used with base 10).

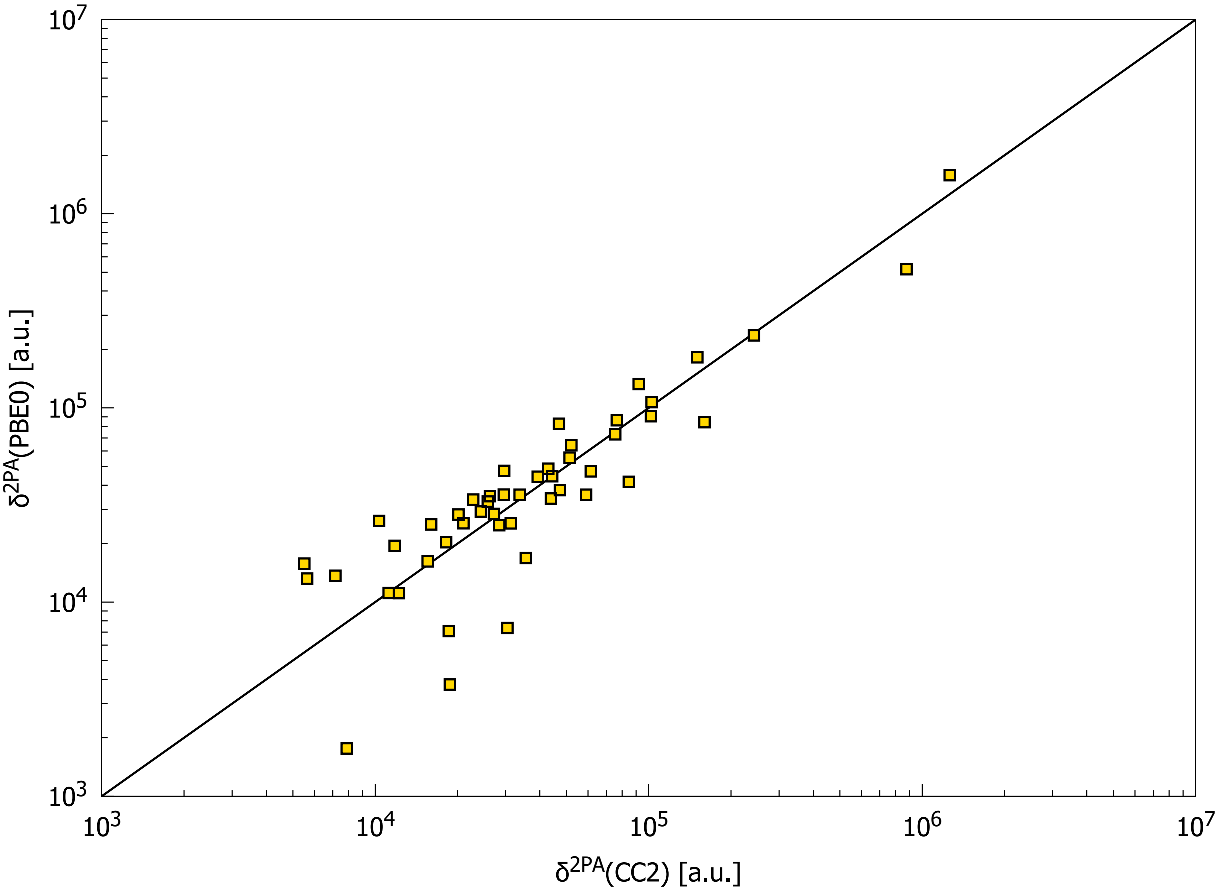

Figure S6: Comparison of two-photon absorption strengths computed using RI-CC2 method and PBE0 functional (double logarithmic scale is used with base 10).

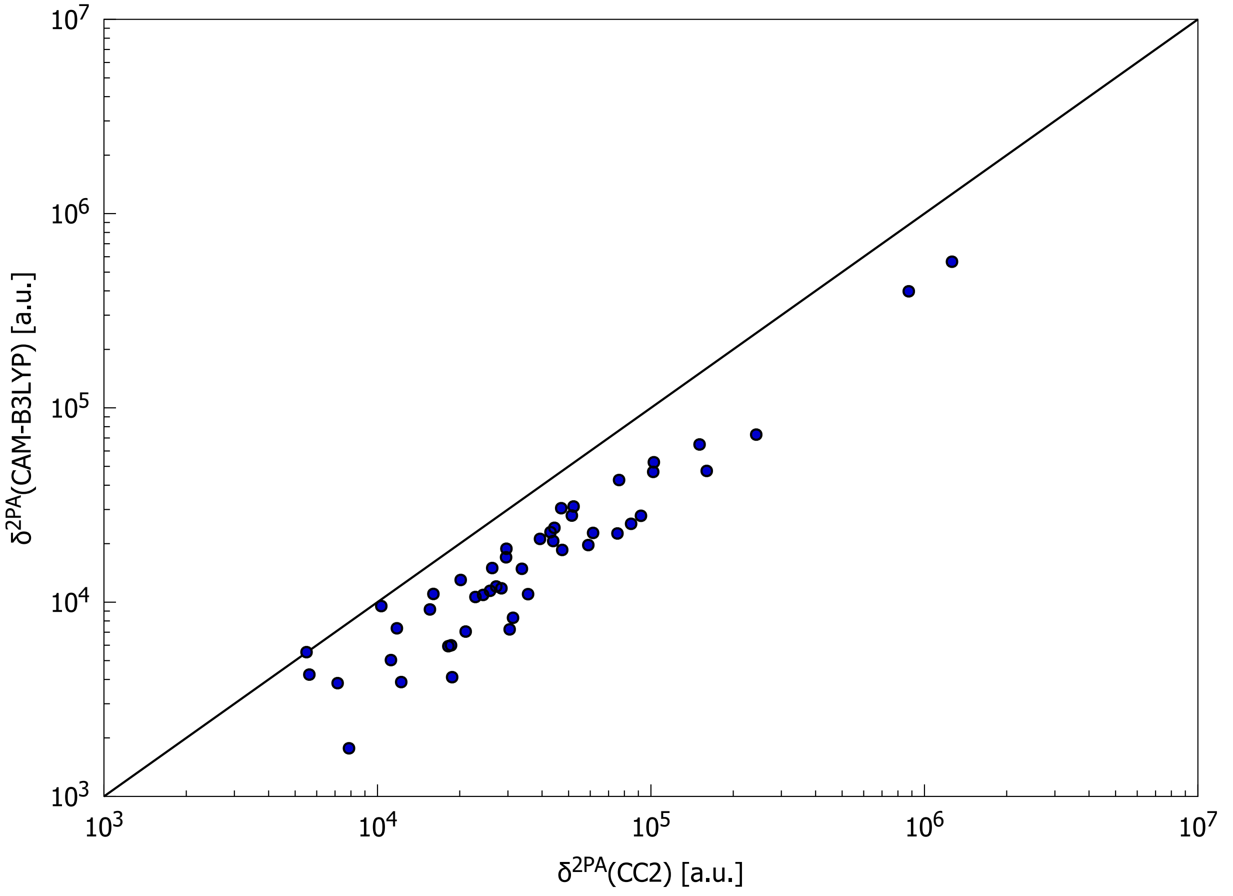

Figure S7: Comparison of two-photon absorption strengths computed using RI-CC2 method and CAM-B3LYP functional (double logarithmic scale is used with base 10).

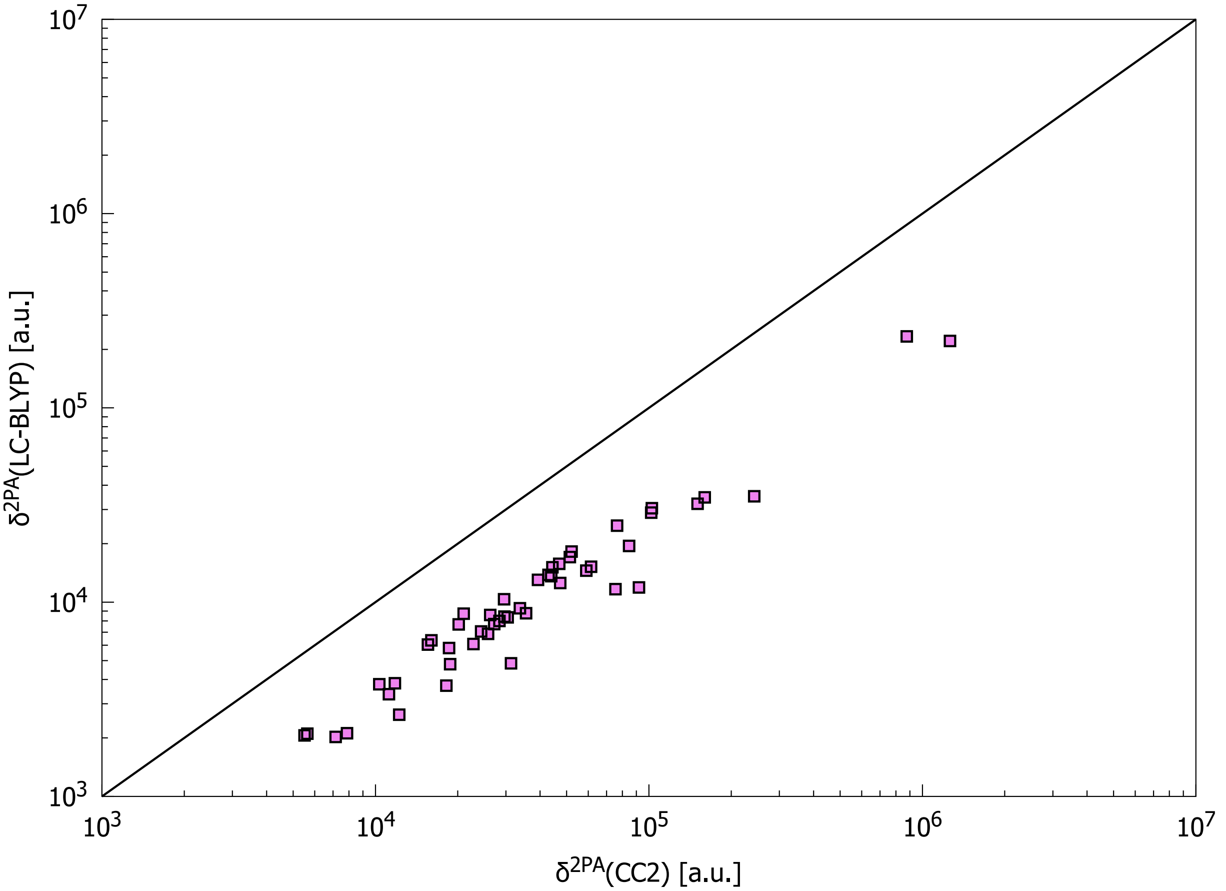

Figure S8: Comparison of two-photon absorption strengths computed using RI-CC2 method and LC-BLYP functional (double logarithmic scale is used with base 10).

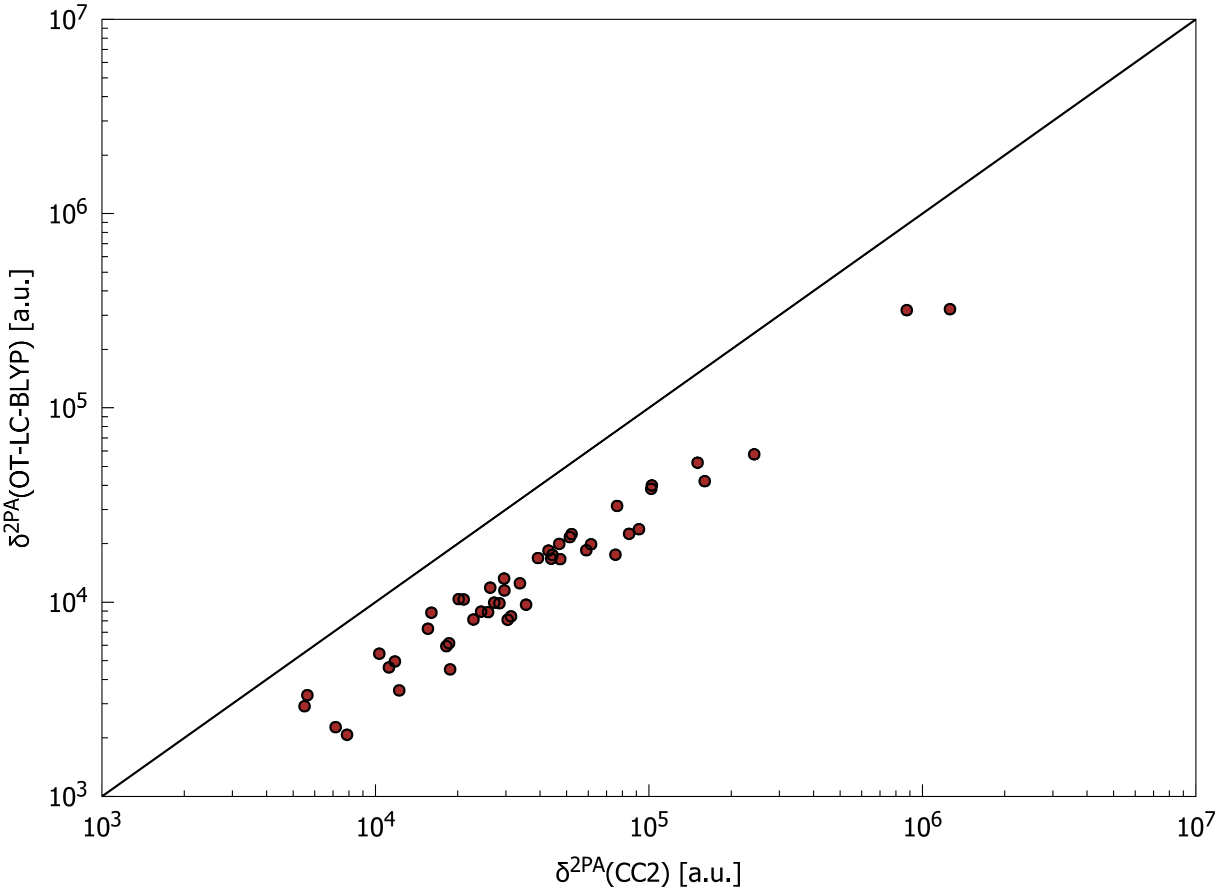

Figure S9: Comparison of two-photon absorption strengths computed using RI-CC2 method and OT-LC-BLYP functional (double logarithmic scale is used with base 10).

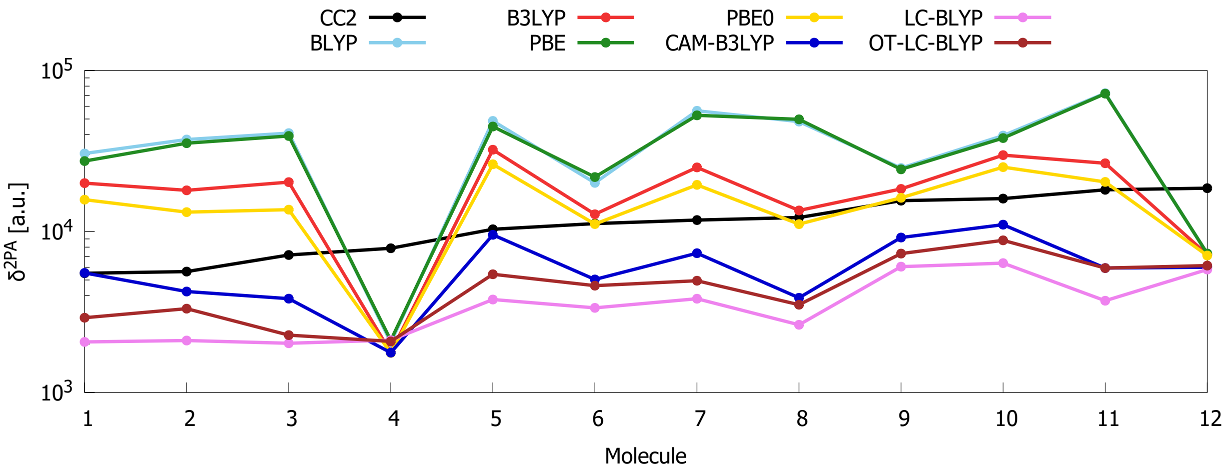

Figure S10: Two-photon absorption strengths computed using RI-CC2 method and density functional approximations for molecules 1-12 (logarithmic scale is used with base 10).

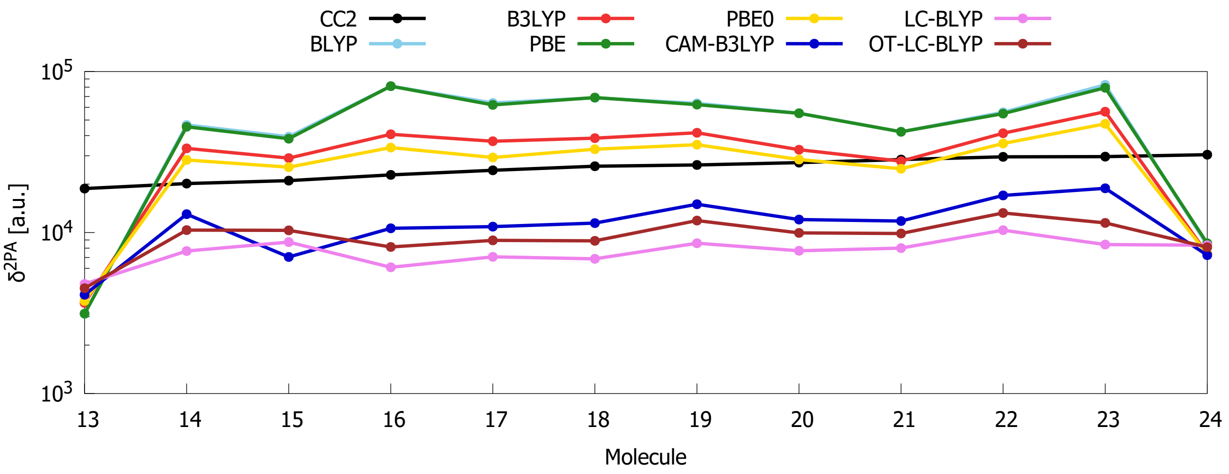

Figure S11: Two-photon absorption strengths computed using RI-CC2 method and density functional approximations for molecules 13-24 (logarithmic scale is used with base 10).

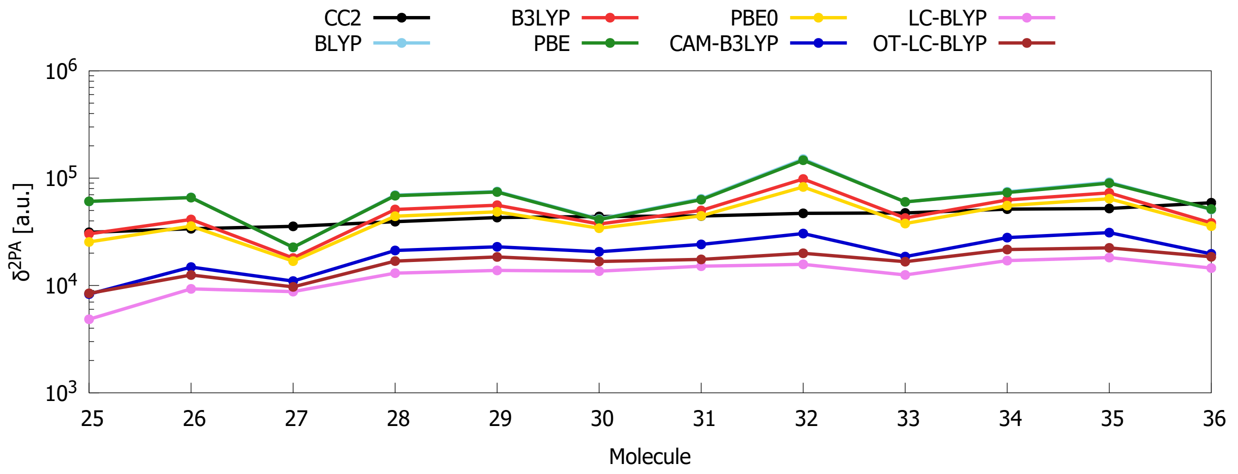

Figure S12: Two-photon absorption strengths computed using RI-CC2 method and density functional approximations for molecules 25-36 (logarithmic scale is used with base 10).

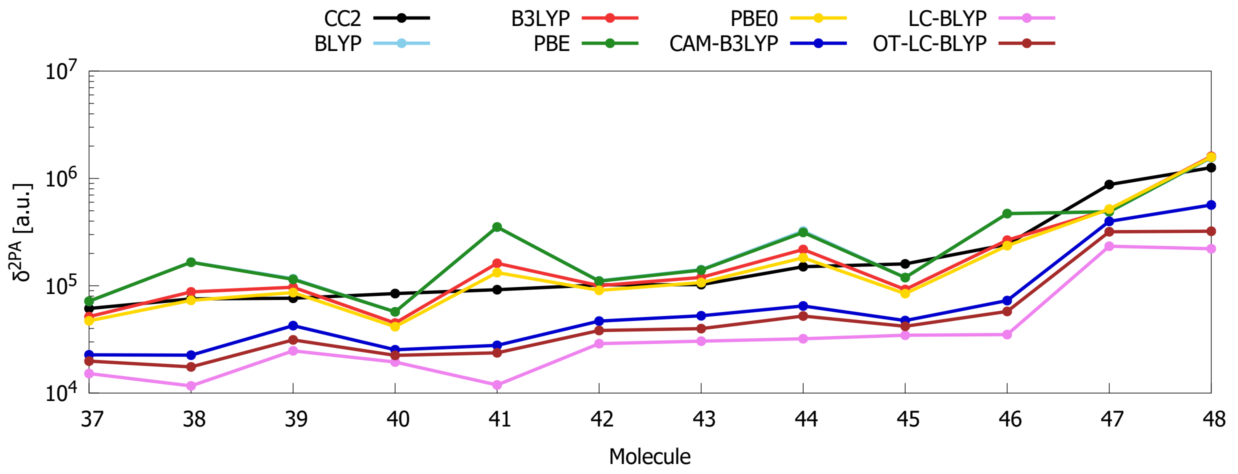

Figure S13: Two-photon absorption strengths computed using RI-CC2 method and density functional approximations for molecules 37-48 (logarithmic scale is used with base 10).

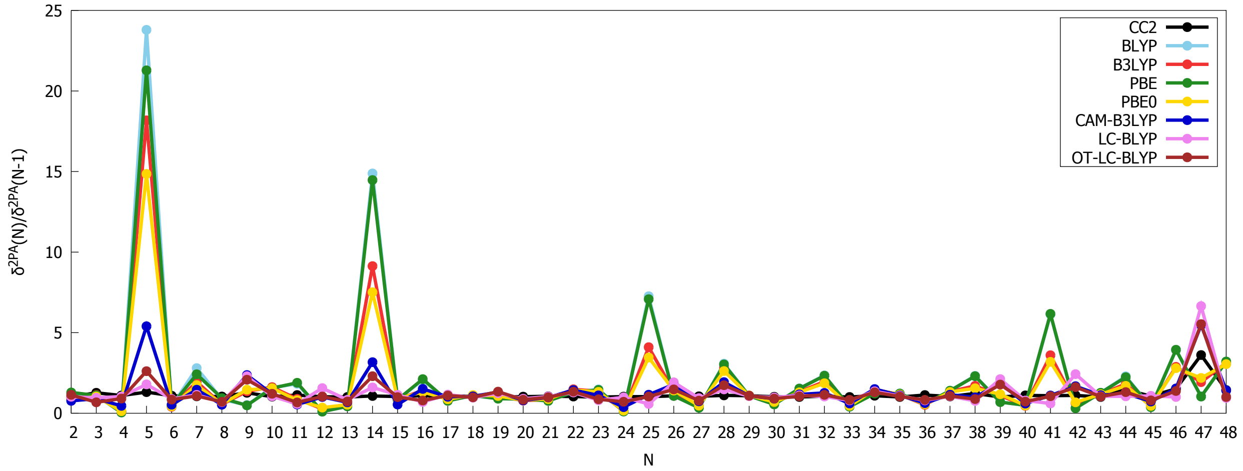

Figure S14: Relative two-photon transition strengths on passing from molecule labelled as $N-1$ to molecule N.

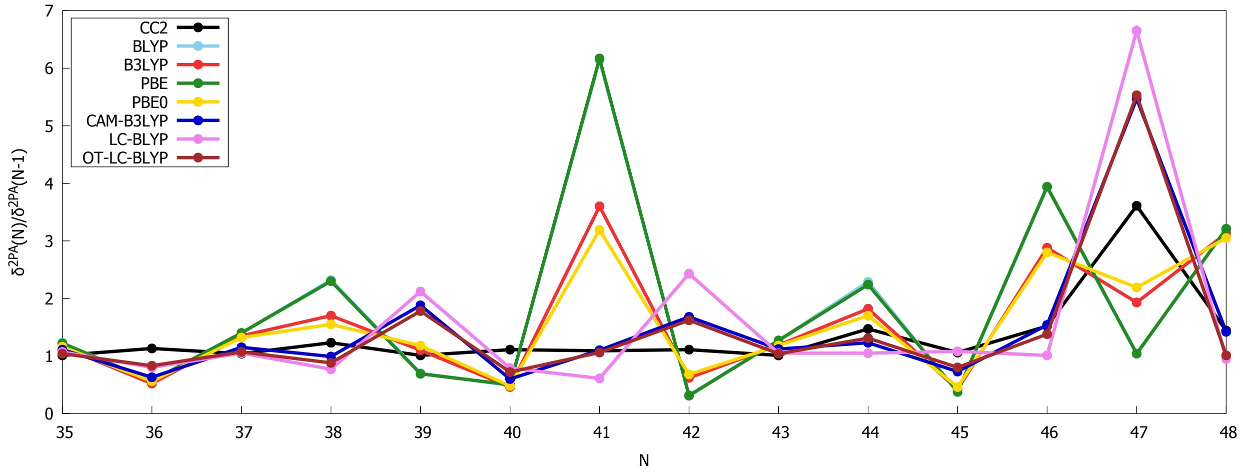

Figure S15: Relative two-photon transition strengths on passing from molecule labelled as $N-1$ to molecule N.

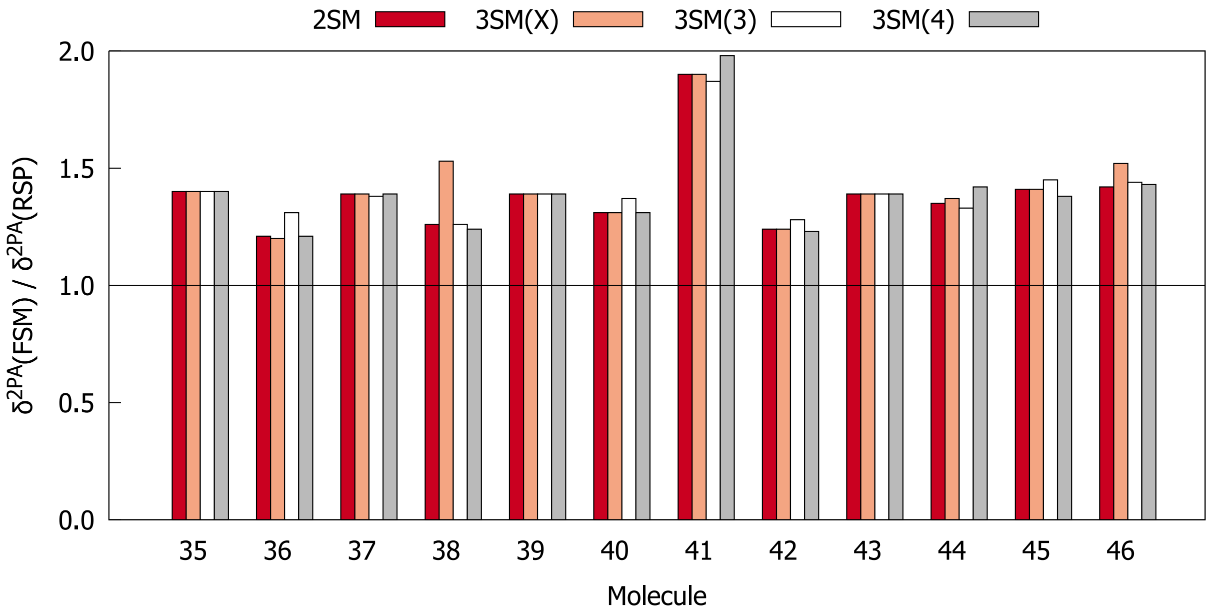

Figure S16: Ratios between the two-photon absorption strengths (δ^{2PA}) computed with few-state models (FSM), i.e. two-state model (2SM) as well as three-state model with additional state 2 (for $S_0 \rightarrow S_1$) or 1 (for $S_0 \rightarrow S_2$), 3 and 4 (3SM(X), 3SM(3), 3SM(4), respectively), and with response theory (RSP) at the RI-CC2/aug-cc-pVDZ level of theory.

Table S1: Two-photon absorption strengths ($\delta^{2PA}(\mathrm{RSP})$, in a.u.) computed with response theory using aug-cc-pVDZ basis set.

	$\delta^{2PA}(\mathrm{RSP})$							
Molecule	CC2	BLYP	B3LYP	CAM-B3LYP	LC-BLYP	PBE	PBE0	OT-LC-BLYP
1	5505	30551	19979	5530	2062	27403	15781	2914
2	5638	37266	18020	4238	2104	35358	13203	3319
3	7149	40865	20277	3830	2024	39225	13668	2273
4	7868	2045	1773	1769	2115	2110	1762	2077
5	10325	48642	32228	9548	3782	44899	26174	5427
6	11206	20063	12803	5037	3357	21790	11131	4610
7	11783	56227	25019	7334	3826	52695	19485	4950
8	12213	48165	13513	3878	2635	49765	11115	3511
9	15552	24794	18385	9178	6051	24346	16206	7297
10	16018	39450	29819	11029	6364	38057	25115	8821
11	18163	72281	26544	5930	3718	71839	20331	5932
12	18581	7288	7192	5998	5807	7299	7092	6144
13	18758	3131	3653	4108	4796	3138	3760	4509
14	20150	46577	33357	13011	7679	45400	28237	10358
15	21020	39403	28997	7054	8726	38275	25484	10319
16	22823	81186	40753	10632	6087	81120	33715	8135
17	24355	63830	36924	10890	7071	62095	29269	8945
18	25813	68704	38565	11445	6866	68927	32932	8883
19	26290	63437	41687	14990	8588	62343	35131	11870
20	27177	55169	32713	12048	7707	55039	28440	9948
21	28407	42228	27767	11789	8008	42285	24896	9864
22	29547	55825	41414	17013	10345	54769	35738	13230
23	29630	82686	56332	18834	8426	79307	47373	11479
24	30443	8353	7378	7237	8347	8579	7355	8117

Table S2: Two-photon absorption strengths ($\delta^{2PA}(\mathrm{RSP})$, in a.u.) computed with response theory using aug-cc-pVDZ basis set.

	$\delta^{2PA}(\mathrm{RSP})$							
Molecule	CC2	BLYP	B3LYP	CAM-B3LYP	LC-BLYP	PBE	PBE0	OT-LC-BLYP
25	31323	60659	30237	8306	4845	60761	25431	8446
26	33752	66196	41201	14842	9299	65858	35733	12509
27	35549	22609	18014	10994	8768	22682	16862	9703
28	39310	69546	51053	21175	13020	68676	44202	16869
29	42931	75162	55962	22915	13820	74248	48597	18433
30	43943	41670	37356	20624	13605	41139	34149	16740
31	44374	64144	49956	24126	15110	63037	44500	17494
32	46973	150595	98155	30467	15746	146905	82830	19951
33	47367	59901	42526	18570	12544	60106	37746	16653
34	51408	74673	62655	27909	17052	73402	55449	21609
35	52131	91656	72882	31102	18216	89833	64245	22410
36	59003	50986	38234	19687	14523	51494	35692	18499
37	61430	71520	51606	22715	15214	71863	47116	19864
38	75464	166032	87692	22563	11670	165322	73129	17547
39	76492	116377	96889	42504	24753	114733	86413	31303
40	84613	57186	44952	25321	19482	57169	41585	22483
41	91958	350913	162041	27828	11911	352837	132797	23732
42	101917	111378	100326	46851	28885	110347	90623	38328
43	102475	141642	119546	52511	30470	140004	107108	39869
44	150580	323700	217859	64848	32081	314167	182279	52208
45	159947	119274	92260	47400	34606	119200	84498	41929
46	242707	470418	265907	72815	35088	469765	236298	57654
47	876640	487498	513440	398131	233363	489970	518158	318539
48	1260912	1559552	1607396	565723	221213	1573695	1580373	321968

Table S3: The assignment of the lowest bright $S_0 \rightarrow S_J$ transition used in two-state model analysis.

	J							
Molecule	CC2	BLYP	B3LYP	CAM-B3LYP	LC-BLYP	PBE	PBE0	OT-LC-BLYP
35	1	1	1	1	2	1	1	1
36	1	1	1	1	1	1	1	1
37	1	1	1	1	1	1	1	1
38	1	1	1	1	1	1	1	1
39	1	1	1	1	1	1	1	1
40	1	1	1	1	1	1	1	1
41	1	1	1	1	1	1	1	1
42	1	1	1	1	1	1	1	1
43	1	1	1	1	1	1	1	1
44	1	1	1	1	1	1	1	1
45	1	1	1	1	1	1	1	1
46	2	1	2	2	2	1	2	2

Table S4: Two-photon absorption strengths (in a.u.) obtained using two-state model (2SM) and aug-cc-pVDZ basis set.

Molecule	CC2	BLYP	B3LYP	CAM-B3LYP	LC-BLYP	PBE	PBE0	OT-LC-BLYP
				δ^{2PA}(2SM)				
35	73140	125918	114601	47996	25601	124324	103397	33068
36	71288	78744	59557	26931	18434	78863	54993	24865
37	85155	110948	86191	33710	20751	110480	78354	29206
38	95228	266301	163730	35301	15453	262607	136200	22056
39	106067	155424	149244	65835	34817	154217	136678	46478
40	111182	98657	75983	37031	26730	98176	69185	32330
41	175075	557043	368527	57937	20057	555030	319260	46153
42	125868	154140	155904	68698	38256	153599	142728	53930
43	142584	187015	183331	82551	43742	185797	169053	60689
44	202639	461948	372347	104776	45552	450863	319317	79376
45	225759	212175	163961	71928	49121	210900	147562	62351
46	345282	727782	363400	117543	49679	723534	381361	90369

Table S5: The percentage contributions of δ_{00}, $\delta_{0J+J0} = \delta_{0J} + \delta_{J0}$ and δ_{JJ} terms to the two-photon absorption strength within a two-state approximation.

	CC2			BLYP			B3LYP			CAM-B3LYP		
Molecule	δ_{00}	δ_{0J+J0}	δ_{JJ}	δ_{00}	δ_{0J+J0}	δ_{JJ}	δ_{00}	δ_{0J+J0}	δ_{JJ}	δ_{00}	δ_{0J+J0}	δ_{JJ}
35	27	-157	230	41	-210	269	32	-177	245	55	-257	302
36	64	-288	324	92	-375	383	99	-396	397	158	-568	510
37	42	-213	271	54	-256	302	57	-265	308	110	-428	419
38	10	-85	174	6	-63	156	7	-69	162	24	-147	223
39	28	-161	233	50	-241	291	35	-188	253	55	-259	304
40	32	-178	246	56	-263	307	65	-291	326	97	-390	393
41	5	-57	152	3	-37	135	3	-39	136	17	-115	198
42	42	-213	271	70	-307	337	50	-241	291	78	-332	354
43	26	-156	229	52	-249	297	34	-186	252	52	-250	297
44	18	-119	201	20	-131	210	15	-109	194	36	-191	255
45	29	-165	236	52	-248	296	60	-274	314	94	-382	388
46	2	-28	126	2	-33	131	2	-32	130	5	-48	143
	LC-BLYP			PBE			PBE0			OT-LC-BLYP		
Molecule	δ_{00}	δ_{0J+J0}	δ_{JJ}	δ_{00}	δ_{0J+J0}	δ_{JJ}	δ_{00}	δ_{0J+J0}	δ_{JJ}	δ_{00}	δ_{0J+J0}	δ_{JJ}
35	84	-351	367	40	-207	266	33	-180	247	72	-312	341
36	195	-668	573	92	-377	385	102	-406	404	172	-606	534
37	154	-555	502	55	-259	304	60	-276	316	128	-482	454
38	45	-222	278	6	-63	157	8	-74	166	34	-185	251
39	86	-357	371	49	-237	288	35	-188	253	72	-314	342
40	109	-425	417	56	-260	305	67	-297	330	105	-415	410
41	41	-210	269	3	-37	135	3	-41	138	22	-137	215
42	111	-433	422	68	-302	334	50	-242	292	93	-379	386
43	82	-344	363	51	-245	294	34	-185	251	67	-298	331
44	63	-284	321	20	-129	209	16	-112	196	45	-226	280
45	107	-421	414	51	-246	294	62	-281	319	105	-414	409
46	7	-63	155	2	-33	131	2	-31	129	5	-44	139

Table S6: The δ_{00} term (in a.u.) contributing to the two-photon absorption strength within a two-state approximation.

	δ_{00}							
Molecule	CC2	BLYP	B3LYP	CAM-B3LYP	LC-BLYP	PBE	PBE0	OT-LC-BLYP
35	19653	51671	36795	26253	21492	49786	33854	23697
36	45567	72155	58754	42645	35860	72932	56164	42790
37	35585	60207	49218	36929	31899	60957	47235	37379
38	9797	16766	12044	8557	6877	16850	11280	7548
39	29447	77561	52299	36394	29863	75048	47822	33477
40	35928	55725	49312	35807	29018	54638	46178	34001
41	9416	14286	10154	9605	8228	14339	9886	10038
42	52630	107819	77632	53362	42462	105078	71586	50129
43	37765	97553	63150	43321	35763	94794	57524	40783
44	35527	93836	57000	37401	28608	89419	51101	36076
45	65015	110203	97975	67684	52637	108044	91262	65196
46	6059	16258	8067	5304	3578	16175	7897	4659

Table S7: The δ_{0J} ($J = 1$ or 2) term (in a.u.) contributing to the two-photon absorption strength within a two-state approximation.

	δ_{0J}							
Molecule	CC2	BLYP	B3LYP	CAM-B3LYP	LC-BLYP	PBE	PBE0	OT-LC-BLYP
35	-57496	-132244	-101648	-61696	-44909	-128372	-92939	-51644
36	-102517	-147497	-117879	-76510	-61545	-148736	-111711	-75380
37	-90631	-141936	-114350	-72211	-57627	-143020	-108070	-70419
38	-40329	-83572	-56441	-25933	-17183	-83356	-50468	-20448
39	-85315	-187333	-140625	-85328	-62098	-182607	-128650	-72910
40	-99100	-129863	-110516	-72210	-56853	-127870	-102693	-67139
41	-50017	-103492	-71324	-33194	-21075	-103547	-66063	-31561
42	-133977	-236711	-187622	-113885	-82742	-232096	-172642	-102098
43	-111145	-232624	-170748	-103123	-75315	-227506	-156138	-90534
44	-120351	-302014	-202664	-99986	-64696	-290183	-178822	-89574
45	-186086	-263098	-224701	-137434	-103453	-258975	-207288	-128919
46	-48510	-120123	-58942	-28177	-15530	-119379	-59276	-20090

Table S8: The δ_{JJ} ($J = 1$ or 2) term (in a.u.) contributing to the two-photon absorption strength within a two-state approximation.

	δ_{JJ}							
Molecule	CC2	BLYP	B3LYP	CAM-B3LYP	LC-BLYP	PBE	PBE0	OT-LC-BLYP
35	168480	338734	281102	145135	93926	331283	255421	112659
36	230754	301584	236562	137306	105665	303404	222252	132836
37	230833	334613	265673	141203	104105	335564	247260	132664
38	166089	416679	264567	78610	42942	412469	225856	55403
39	247251	452528	378196	200098	129150	444382	346155	158822
40	273453	302658	247704	145645	111419	299278	228392	132606
41	265694	749740	501022	114720	53978	747786	441501	99238
42	341192	519742	453516	243107	161278	512712	416427	207997
43	327108	554709	461677	245475	158609	546015	423804	200972
44	407814	972140	720675	267347	146336	941811	625859	222448
45	532916	628168	515389	279111	203390	620806	470876	254993
46	436243	951771	473217	168593	77160	946118	492016	125889

Table S9: The ground-state dipole moment ($|\mu_{00}|$, in a.u.) computed using DFT and CC2 method and the aug-cc-pVDZ basis set.

				$|\mu_{00}|$				
Molecule	CC2	BLYP	B3LYP	CAM-B3LYP	LC-BLYP	PBE	PBE0	OT-LC-BLYP
35	3.0854	3.8605	3.6706	3.4263	3.2860	3.8404	3.6181	3.3133
36	3.7855	4.1924	4.0204	3.8145	3.7189	4.2209	4.0047	3.7959
37	3.6959	4.1488	3.9785	3.7839	3.7006	4.1777	3.9640	3.7627
38	1.8175	2.0037	1.8748	1.7536	1.7097	2.0042	1.8493	1.7517
39	3.4757	4.3022	4.0671	3.7840	3.6343	4.2833	4.0068	3.6731
40	2.8310	3.1727	3.0936	2.9031	2.7704	3.1536	3.0502	2.8042
41	1.7075	1.9141	1.7867	1.6724	1.6302	1.9122	1.7606	1.6650
42	3.8271	4.6054	4.3414	4.0169	3.8471	4.5872	4.2735	3.9134
43	3.7011	4.5241	4.2612	3.9579	3.8068	4.5077	4.1970	3.8546
44	2.8992	3.6636	3.4060	3.1057	2.9369	3.6145	3.3240	2.9979
45	3.0611	3.5398	3.4150	3.1508	2.9758	3.5177	3.3575	3.0442
46	1.2274	1.5867	1.4189	1.2218	1.1082	1.5848	1.3907	1.1485

Table S10: The right and left transition moment product ($|\mu_{0J*J0}| = \sqrt{\mu_x^{0J}\mu_x^{J0} + \mu_y^{0J}\mu_y^{J0} + \mu_z^{0J}\mu_z^{J0}}$, $J = 1$ or 2, in a.u.) computed using DFT and CC2 method and the aug-cc-pVDZ basis set.

	$\|\mu_{0J*J0}\|$							
Molecule	CC2	BLYP	B3LYP	CAM-B3LYP	LC-BLYP	PBE	PBE0	OT-LC-BLYP
35	3.6040	2.7834	3.1228	3.5395	3.5854	2.7903	3.2264	3.5956
36	3.9713	3.5285	3.8268	3.9117	3.8434	3.5282	3.8773	3.8758
37	3.9450	3.3263	3.7023	3.9159	3.9071	3.3242	3.7691	3.9570
38	3.7090	3.0428	3.4895	3.8475	3.7422	3.0549	3.5880	3.4787
39	3.7305	2.8797	3.2008	3.6626	3.7274	2.8867	3.3070	3.7321
40	4.6256	4.0650	4.4605	4.5727	4.5045	4.0623	4.5125	4.5593
41	3.9838	2.6562	3.2565	4.3326	4.3667	2.6657	3.4601	4.3435
42	4.0215	3.0751	3.4858	3.9017	3.8930	3.0872	3.6015	3.9099
43	3.8191	2.9345	3.2455	3.7510	3.8391	2.9413	3.3540	3.8354
44	4.0864	3.1242	3.5175	4.1222	4.1152	3.1470	3.6615	4.1477
45	5.4245	4.6477	5.2211	5.3851	5.2966	4.6435	5.2915	5.3870
46	4.3795	3.3553	3.5849	4.4922	4.4838	3.3676	3.8487	4.5320

Table S11: The excited state dipole moment ($|\mu_{JJ}|$, $J = 1$ or 2, in a.u.) computed using DFT and CC2 method and the aug-cc-pVDZ basis set.

	$\lvert\mu_{JJ}\rvert$							
Molecule	CC2	BLYP	B3LYP	CAM-B3LYP	LC-BLYP	PBE	PBE0	OT-LC-BLYP
35	9.0048	9.8660	10.1230	8.0355	6.8511	9.8878	9.9155	7.2054
36	8.5132	8.5661	8.0618	6.8389	6.3781	8.6041	7.9608	6.6830
37	9.4133	9.7808	9.2433	7.3990	6.6852	9.8019	9.0693	7.0887
38	7.4602	9.9764	8.7708	5.3027	4.2617	9.9033	8.2588	4.7394
39	10.0647	10.3881	10.9322	8.8683	7.5537	10.4189	10.7750	7.9963
40	7.8021	7.3913	6.9305	5.8501	5.4223	7.3774	6.7796	5.5322
41	9.0694	13.8659	12.5498	5.7790	4.1743	13.8086	11.7647	5.2347
42	9.7373	10.1075	10.4875	8.5675	7.4914	10.1288	10.3012	7.9656
43	10.8926	10.7881	11.5215	9.4214	8.0168	10.8185	11.3919	8.5568
44	9.8180	11.7890	12.1064	8.2987	6.6382	11.7276	11.6281	7.4403
45	8.7474	8.4466	7.8269	6.3901	5.8386	8.4267	7.6201	6.0110
46	9.2283	11.2022	9.7952	6.0168	4.3999	11.1684	9.8373	5.1643

Table S12: The excitation energy (ΔE_{0J}, $J = 1$ or 2, in a.u.) computed using DFT and CC2 method and the aug-cc-pVDZ basis set.

	ΔE_{0J}							
Molecule	CC2	BLYP	B3LYP	CAM-B3LYP	LC-BLYP	PBE	PBE0	OT-LC-BLYP
35	0.14138	0.08438	0.10662	0.13348	0.14326	0.08572	0.11320	0.13798
36	0.12590	0.09844	0.11344	0.12911	0.13487	0.09858	0.11709	0.12711
37	0.13826	0.10061	0.11877	0.13793	0.14481	0.10062	0.12297	0.13776
38	0.12094	0.08404	0.10616	0.12933	0.13649	0.08419	0.11119	0.12420
39	0.13505	0.07954	0.10177	0.12987	0.14011	0.08070	0.10833	0.13392
40	0.12342	0.09765	0.11103	0.12526	0.13078	0.09795	0.11442	0.12381
41	0.12538	0.07609	0.10328	0.13221	0.14031	0.07615	0.10959	0.12909
42	0.11991	0.07711	0.09708	0.12124	0.12987	0.07811	0.10282	0.12213
43	0.13011	0.07604	0.09845	0.12759	0.13824	0.07703	0.10499	0.13096
44	0.11238	0.06682	0.08972	0.11833	0.12771	0.06803	0.09626	0.11703
45	0.11618	0.08853	0.10169	0.11628	0.12241	0.08876	0.10496	0.11451
46	0.10924	0.06881	0.09112	0.11713	0.12590	0.06905	0.09634	0.11584

Table S13: The minimum, maximum and average values over $|1-\mathrm{A(DFT)/A(CC2)}|$ terms, where A is δ_{00}, δ_{0J}, δ_{JJ}, $|\mu_{00}|$, $|\mu_{0J*J0}|$, $|\mu_{JJ}|$ or ΔE_{0J}, obtained for molecules 35-46.

	BLYP	B3LYP	CAM-B3LYP	LC-BLYP	PBE	PBE0	OT-LC-BLYP
				δ_{00}			
min	0.517	0.078	0.003	0.014	0.521	0.050	0.003
max	1.683	0.872	0.336	0.409	1.669	0.723	0.231
ave	1.081	0.466	0.100	0.173	1.043	0.368	0.098
				δ_{0J}			
min	0.310	0.115	0.000	0.219	0.290	0.036	0.102
max	1.509	0.768	0.419	0.680	1.461	0.616	0.586
ave	0.934	0.401	0.214	0.427	0.906	0.294	0.291
				δ_{JJ}			
min	0.107	0.025	0.139	0.443	0.094	0.037	0.331
max	1.822	0.886	0.614	0.823	1.814	0.662	0.711
ave	0.833	0.381	0.388	0.606	0.812	0.292	0.484
				$\|\mu_{00}\|$			
min	0.102	0.031	0.005	0.001	0.103	0.017	0.003
max	0.293	0.190	0.110	0.097	0.291	0.173	0.074
ave	0.183	0.117	0.045	0.036	0.180	0.101	0.032
				$\|\mu_{0J*J0}\|$			
min	0.111	0.036	0.007	0.001	0.112	0.024	0.000
max	0.333	0.183	0.088	0.096	0.331	0.131	0.090
ave	0.203	0.108	0.024	0.023	0.201	0.079	0.024
				$\|\mu_{JJ}\|$			
min	0.006	0.018	0.108	0.231	0.007	0.037	0.182
max	0.529	0.384	0.363	0.540	0.523	0.297	0.440
ave	0.132	0.124	0.214	0.331	0.132	0.108	0.278
				ΔE_{0J}			
min	0.209	0.099	0.001	0.013	0.206	0.070	0.003
max	0.416	0.246	0.072	0.153	0.408	0.199	0.060
ave	0.333	0.171	0.035	0.080	0.329	0.129	0.021
